# Atropisomers of *meso* Tetra(*N*‐Mesyl Pyrrol‐2‐yl) Porphyrins: Synthesis, Isolation and Characterization of All‐Pyrrolic Porphyrins

**DOI:** 10.1002/chem.201905637

**Published:** 2020-03-06

**Authors:** Leiming Zhu, Leonard Himmel, Jean Michél Merkes, Fabian Kiessling, Magnus Rueping, Srinivas Banala

**Affiliations:** ^1^ Institute for Organic Chemistry RWTH Aachen University Landoltweg 1 52074 Aachen Germany; ^2^ Institute for Experimental Molecular Imaging, Uniclinic RWTH Aachen University Forckenbeckstr 55 52074 Aachen Germany; ^3^ KAUST Catalysis Center (KCC) King Abdullah University of Science and Technology (KAUST) Thuwal 23955-6900 Saudi Arabia

**Keywords:** atropisomers, *meso* tetra(pyrrol-2-yl) porphyrin, porphyrinoids absorption spectra, porphyrins, transition-metal complexes

## Abstract

Atropisomerism has been observed in a variety of biaryl compounds and *meso*‐aryl substituted porphyrins. However, in porphyrins, this phenomenon had been shown only with *o*‐substituted 6‐membered aromatic groups at the *meso*‐position. We show herein that a 5‐membered heteroaromatic (*N*‐mesyl‐pyrrol‐2‐yl) group at the *meso*‐position leads to atropisomerism. In addition, we report a ‘one‐pot’ synthetic route for the synthesis of ‘all‐pyrrolic’ porphyrin (APP) with several N‐protection groups (Boc, Cbz, Ms and Ts). Among these groups, we found that only the Ms group gave four individually separable atropisomers of *meso*‐tetra(*N*‐Ms‐pyrrol‐2‐yl) porphyrin. Furthermore, the reductive removal of Cbz‐ was achieved to obtain *meso*‐tetra(pyrrol‐2‐yl) porphyrin. Thus, our synthetic procedure provides an easy access to a group of APPs and stable atropisomers, which is expected to expand the application of novel APP‐based materials.

## Graphical abstract

**Stable and separable** atropisomers of *meso* tetra(*N*‐mesyl pyrrol‐2‐yl) porphyrin were synthesized. Other N‐protection groups (*tert*‐butyloxycarbonyl (Boc), carboxybenzyl (Cbz), and Tosyl (Ts)) were explored in the ‘all‐pyrrolic porphyrins’ synthesis, but only a mixture of inseparable atropisomers were obtained.

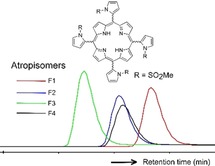

## Introduction

**Figure 1:**
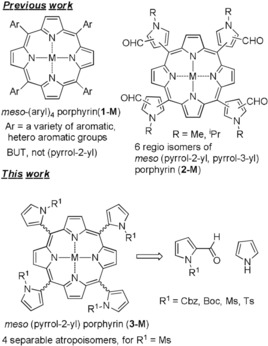
Structures of *meso*‐tetra aryl (**1**) and (pyrrolyl) porphyrins (**2**, **3**).

Porphyrin chemistry has been vastly developed over the last decades, as the tetrapyrroles in hemin, chlorophyll, and vitamin B12, the ‘Pigments of Life’,^1^ were valuable targets for synthetic, biomimetic, and therapeutic applications. The conjugated, macrocyclic tetrapyrroles exhibit strong electronic absorption in the visible range and are highly stable, making them attractive, for biomedicine,^2^ catalysis,^3^ materials,^4^ and electronics.^5^ To fine‐tune the photophysical properties, modifications in the *meso*‐position with aryl, heteroaryl, and alkynyl groups (e.g. **1‐M**, Figure 1) have been widely used.4c, 6 This included symmetric and mixed substituents (so called, A3B‐, A2B2‐, A2BC‐, and ABCD‐types) of porphyrins.^7^ In this process, by employing *ortho*‐substituted 6‐membered aromatic groups at *meso*‐positions, which hinder rotation around the plane of porphyrins, atropisomers were obtained.^8^ These atropisomeric porphyrins were widely studied as models for bioinorganic, and applied as ligands,^9^ especially attached with chiral moieties,^10^ in catalyst development.^11^

Although porphyrin synthesis is highly matured, surprisingly ‘all‐pyrrolic’ *meso*‐(pyrrolyl) porphyrins (APPs, for example, **2**, **3**) are seldom found in the literature.^12^ In addition, the influence of N‐substituents on pyrrol‐2‐yl porphyrin, and the possible formation of atropisomers, by such substituents are unknown. Already outside of porphyrin chemistry,^13^ stable atropisomers of *N*‐aryl pyrroles were reported,^14^ in which the pyrrole was substituted at the 2‐position or at the N atom with an *ortho*‐aryl group.^15^ Similarly, 3‐aryl pyrrole with an additional substituent at the 4‐position yielded separable atropisomers.^16^ Thus, we presumed that incorporating N‐protected pyrroles in a porphyrin at *meso*‐position (i.e. *meso*‐(pyrrol‐2‐yl) porphyrin) could yield separable atropisomers, and initiated the synthesis of APPs with different N‐protection groups.

Despite the availability of several synthetic procedures^17^ and a variety of *meso*‐aryl substituted porphyrins,^18^ APP is a challenging target. To date, only one synthesis of APP has been reported, using an *N*‐alkyl group (**2‐M**, Figure 1, top).^12^ For its preparation, N‐alkylated (Me, *i*Pr) 2,4‐diformyl pyrrole was treated with pyrrole in the presence of acid catalysts, and six regioisomers of APP could be isolated from the mixture, but no atropisomers.^12^ Similarly, no atropisomers were found for mono‐19 and bis‐*meso* (pyrrol‐2‐yl) porphyrins,^20^ albeit using 1*H*‐pyrrole.

We are interested in exploring a suitable ‘one‐pot’ method for the synthesis of tetra(*N*‐protected pyrrol‐2‐yl) porphyrins. In addition, by choosing a variety of N‐protection groups for the pyrrole‐2‐aldehyde, we wished to explore the formation of atropisomers, and their isolation. Herein, we report successful procedures towards those APPs, and the isolation and characterization of stable atropisomers.

## Results and Discussion

For the synthesis of APP **3**, we envisaged to apply ‘one‐pot’ tetramerization procedures. Among several known methods, we chose to explore NH_2_OH**⋅**HCl mediated condensation, as it was successfully applied to *meso* tetra(theinyl‐2‐yl) porphyrin synthesis.^21^ In addition, the broadly applicable Lindsey's method, using catalytic BF_3_ **⋅**OEt_2_,^18^ and a recently reported reaction using *p*‐toluenesulfonic acid (pTSA) in hot DMF were selected.^22^ As in the synthesis of **2**, N‐alkylated (Me, *i*Pr) pyrrole‐2‐aldehydes (**4**‐Me, **4**‐*i*Pr) were explored in former two conditions. However, they did not yield any product, and only starting materials were observed. We assumed that the Lewis acid activated formyl group was stabilized by the electron‐rich nature of pyrrole, suppressing further reaction towards the condensation. Thus, we anticipated that removal of electron density from the pyrrole‐2‐aldehyde could enhance the tendency for the condensation reaction.

**Scheme 1:**
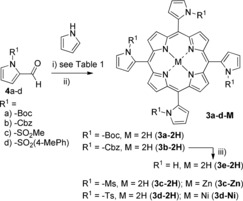
Tetramerization of pyrrol‐2‐aldehydes and pyrrole towards **3‐M**. i) Conditions in Table 1 and Table S2; ii) in DMF, 100 °C; for **3‐Zn**, Zn(OAc)_2_ and for **3‐Ni**, Ni(OAc)_2_. iii) THF/MeOH (4:1), 5 % Pd/C, H_2_ (1 atm), rt.

Therefore, we introduced electron‐withdrawing groups at the nitrogen of the pyrrole‐2‐aldehyde. We chose commonly used protection groups; *tert*‐butyloxycarbonyl (Boc, **4 a**), carboxybenzyl (Cbz, **4 b**), methanesulfonyl (Ms, **4 c**) and tosyl (Ts, **4 d**), which can be removed under acidic, neutral, and basic conditions, respectively (Scheme 1)

First the NH_2_OH**⋅**HCl‐mediated reactions of **4 a**–**d** with pyrrole were carried out and APPs **3 a**–**d‐2H** could be obtained in 12 to 48 % yields (Table 1). Similarly, Lindsey's method using BF_3_ **⋅**OEt_2_ gave us 11–50 % of APPs. Among the four N‐substituents, Cbz‐protected **4 b** in Lindsey's method gave the highest yield of **3 b‐2H**, 50 %, followed by NH_2_OH**⋅**HCl with Ms‐protected **4 c** giving 48 % of **3 c‐2H**. When **4 d** (Ts‐protected) was employed in the synthesis, 15 and 27 % of **3 d**‐**2H** were isolated, respectively. Other explored conditions with **4 c** and **4 d** gave far lower yields of APPs (Table S2, Supporting Information).

**Table 1:** Tetramerization conditions and obtained yields.

No.	(*N‐*R^1^‐Py‐CHO)	Tetramerization Catalyst	Product (**3R‐2H**)	Yield [%]^[c]^
1	Boc **(4 a)**	NH_2_OH**⋅**HCl^[a]^	**3 a‐2H**	12
2	Boc **(4 a)**	BF_3_ **⋅**OEt_2_^[b]^	**3 a‐2H**	11
4	Cbz **(4 b)**	NH_2_OH**⋅**HCl^[a]^	**3 b‐2H**	10
4	Cbz **(4 b)**	BF_3_ **⋅**OEt_2_^[b]^	**3 b‐2H**	50
5	Ms **(4 c)**	NH_2_OH**⋅**HCl^[a]^	**3 c‐2H**	48
6	Ms **(4 c)**	BF_3_ **⋅**OEt_2_^[b]^	**3 c‐2H**	23
7	Ts **(4 d)**	NH_2_OH**⋅**HCl^[a]^	**3 d‐2H**	27
8	Ts **(4 d)**	BF_3_ **⋅**OEt_2_^[b]^	**3 d‐2H**	15

[a] i) Equimolar amounts of NH_2_OH**⋅**HCl, pyrrole and **4‐R^1^** in chlorobenzene, 24 h, rt. ii) Nitrobenzene, 2 h, 130 °C. [b] i) 0.1 equiv. of BF_3_ **⋅**OEt_2_, into equimolar amounts of pyrrole and **4‐R^1^** in DCM. ii) *p*‐Chloranil, reflux, 1 h. [c] Combined yield of all atropisomers.

To our satisfaction, the *N*‐sulfonyl pyrrole aldehydes (Ms, **4 c** and Ts, **4 d**) yielded chromatographically separable atropisomer mixtures. In particular, the Ms‐protection gave four separable atropisomers of (*N*‐Ms pyrrole‐2‐yl) porphyrin **3 c‐2H** (Figure 3) in 48 % combined yield. With Ts‐protection only one out of four atropisomers could be separated, and the others could only be obtained as a mixture. In contrast, the *N*‐carbamate‐protected pyrrole (Boc, **4 a** and Cbz, **4 b)** gave no separable atropisomers of APP (**3 a‐2H, 3 b‐2H)**, although their existence was proved by NMR spectroscopy.

**Figure 2:**
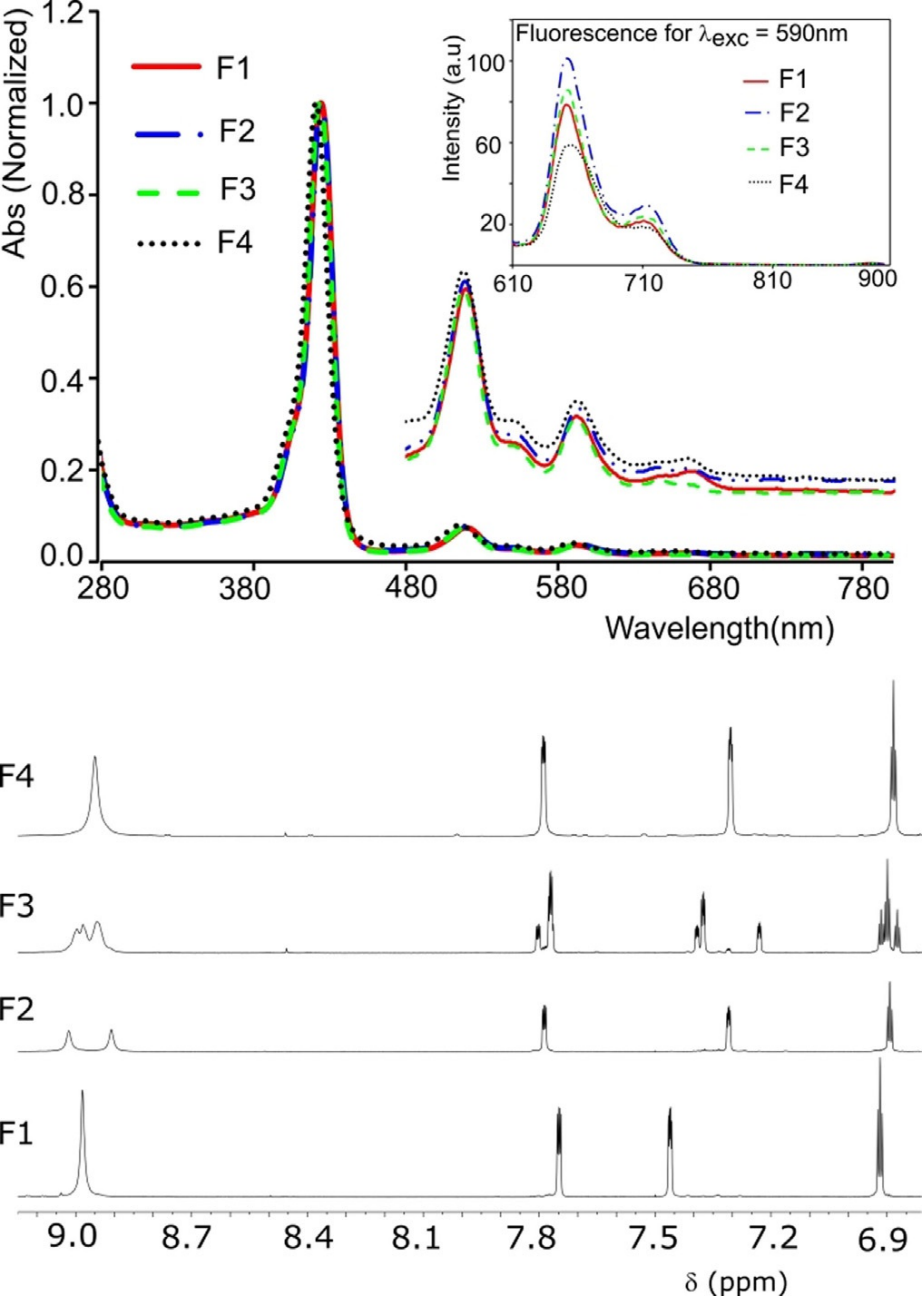
Top: Normalized UV/Vis spectral overlay of **3 c‐2H** atropisomer fractions and fluorescence emission spectra (inset box) for excitation at 590 nm (in CH_2_Cl_2_). Bottom: Part of ^1^H NMR (600 MHz, CD_2_Cl_2_) spectra of atropisomers (see the Supporting Information for full spectra, Figures S17, S19, S21, and S23).

**Figure 3:**
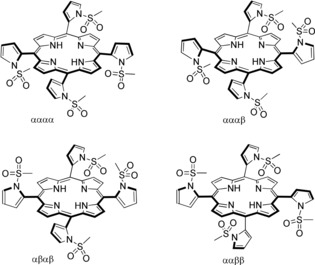
Atropisomers of *meso* (*N*‐mesyl‐pyrrol‐2‐yl) porphyrins **3 c‐2H**.

All four atropisomeric porphyrins **3 c‐2H** exhibited similar UV/Vis absorption characteristics, a Soret band at ≈425 nm and Q bands at ≈517, 554, 591 and 645 nm (Figure 2 top, Table S3, Supporting Information). By using ^1^H NMR, the four atropisomers (Figure 2 bottom, Figures S17—S26, Supporting Information) could be identified: the first nonpolar isomer (F1) gave a highly symmetric spectrum, a *s* for the CH of porphyrin at *δ*=8.98 ppm, hence identified as αααα‐**3 c**‐**2H** (Figure 3). The medium polar fraction (F2) gave two *s* for CH‐protons, hence the αβαβ‐isomer, the following fraction (F3) gave a highly unsymmetrical spectrum, likely corresponding to the ααββ‐isomer. The final polar fraction (F4) showed a symmetric (F1‐like) spectrum, with a broadened *s* CH signal of the porphyrin at *δ*=8.95 ppm (width=36 Hz in 600 NMR); this lead us to assign it as an αααβ‐isomer. The ratio of atropisomers, based on the isolated yields, was found to be 5.9:3.7:2.9:1 (F1:F2:F3:F4).

The stability of the atropisomers was further investigated by variable temperature (VT) NMR in toluene‐d6 (Figures S27 to S30, Supporting Information). Heating the solutions up to 80 °C rendered no change in their respective signal pattern (Figures S26–S29). When the F3 was further heated to 100 °C, for 4 h, some isomerization was observed (Figures S16 and S30, Supporting Information). This confirms that the atropisomers of **3 c‐2H** are highly stable and possess a high isomerization energy barrier.

**Figure 4:**
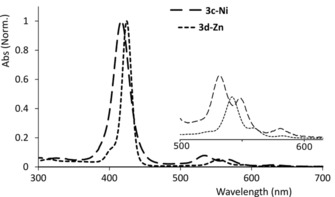
UV/Vis absorption of metallated *meso* (*N*‐sulfonyl 2‐pyrrolyl) porphyrin (inset Q bands).

In the next step, to prove the inherent metal‐complexation ability of APP, *N*‐sulfonyl (pyrrolyl‐2‐yl) porphyrins (**3 c‐2H, 3 d‐2H)** were metallated with Zn^II^ and Ni^II^ acetate in hot DMF. The resulting metallo‐APPs showed the characteristic Q band peaks at 554 nm with a shoulder at 590 nm for **3 c‐Zn**, and at 534 nm with a shoulder at 567 nm for **3 c‐Ni** (Figure 4).

With metallated **3 c‐Zn**, **3 c‐Ni** and **3 d‐Zn**, **3 d‐Ni** in hand, deprotection was carried out using NaOH in THF/MeOH (4:1) and CH_2_Cl_2_/MeOH (5:1). The reactions were stirred at 20 °C under protection from light for up to 72 h; however, no reaction was observed. By refluxing the same mixture, a greenish product was obtained. ^1^H NMR (in CD_2_Cl_2_) of the dry mixtures indicated the formation of a polymeric material. Even under exclusion of oxygen the same results were obtained.

To further explore the feasibility of deprotection of APPs under neutral conditions, *N*‐Cbz removal in **3 b‐2H** (using a mixture of inseparable atropisomers) was studied using 5 % Pd/C and H_2_ (1 atm.) in THF/MeOH. From this we could identify highly symmetric *meso*‐tetra(pyrrol‐2‐yl) porphyrin (**3 e‐2H)**, albeit isolated in protonated form (Figure S36, Supporting Information). However, directly after removing the solvents, no protonation was observed in the reaction mixture as confirmed by ^1^H NMR (CD_3_OD) (Figure S35, Supporting Information). Therefore, the protonation might have occurred during the purification using CH_2_Cl_2_/MeOH. Further spontaneous polymerization of unprotonated electron‐rich **3 e‐2H** was evaluated in an oxygen environment by ^1^H NMR (in CD_3_OD). The **3 e‐2H** was found to be stable even after storing the solution for 90 days at room temperature; however, the polymerization was observed when it was heated. Therefore, selective polymerization to make size‐specific polymeric APP is possible using tetra(pyrrol‐2‐yl) porphyrin, which is currently being explored.

## Conclusions

Herein, we report generally applicable synthetic methods for the preparation of different *meso* tetra(N‐protected pyrrol‐2‐yl) porphyrins (APPs). It was found that an electron‐withdrawing group at the N‐position in pyrrol‐2‐aldehyde was essential for tetramerization to yield APPs. Among the explored conditions, the Lindsey's method with *N*‐Cbz pyrrol‐2‐aldehyde gave 50 %, and the NH_2_OH**⋅**HCl mediated condensation with *N*‐Ms pyrrol‐2‐aldehyde gave 48 % of respective APPs. In addition, the *N*‐Ms pyrrol‐2‐aldehyde gave stable and separable four atropisomers of tetra(*N*‐Ms pyrrol‐2‐yl) porphyrin. These hitherto inaccessible all‐pyrrolic porphyrins were metallated with transition metals. The metallo APPs possess similar photophysical characteristics to *meso* tetra aryl porphyrins. The high reactivity of the *meso* (pyrrol‐2‐yl) group in porphyrin, upon removal of protection group, is a useful feature for the preparation of pyrrole–pyrrole‐bridged porphyrin sheets and nanoparticles, which is currently under investigation.

## Conflict of interest

The authors declare no conflict of interest.

## Supporting information

As a service to our authors and readers, this journal provides supporting information supplied by the authors. Such materials are peer reviewed and may be re‐organized for online delivery, but are not copy‐edited or typeset. Technical support issues arising from supporting information (other than missing files) should be addressed to the authors.

Supplementary
