# Supplementary material for: Atropisomers of meso Tetra(N‐Mesyl Pyrrol‐2‐yl) Porphyrins: Synthesis, Isolation and Characterization of All‐Pyrrolic Porphyrins
Source: Chemistry. 2020 Mar 6;26(19):4232–5. doi: 10.1002/chem.201905637 (PMC7187276; doi:10.1002/chem.201905637)
Supplement: Supplementary file 1 — Supplementary [file CHEM-26-4232-s001.pdf]

CHEMISTRY
A European Journal

Supporting Information

Atropisomers of *meso* Tetra(*N*-Mesyl Pyrrol-2-yl) Porphyrins: Synthesis, Isolation and Characterization of All-Pyrrolic Porphyrins

Leiming Zhu,[a] Leonard Himmel,[a] Jean Michél Merkes,[a] Fabian Kiessling,[b] Magnus Rueping,[a, c] and Srinivas Banala*[a, b]

chem_201905637_sm_miscellaneous_information.pdf

Contents:

1. General S2

2. Experimental procedures for *meso* (pyrrol-2-yl) porphyrins synthesis S2

3. Analytical data for Compounds S5

4. UV-Vis and Fluorescence spectra S7

5. HPLC S11

6. NMR spectra

A) *meso* (*N*-mesyl pyrrol-2-yl) porphyrin (**3c-2H**) S16

B) *meso* (*N*-Boc-pyrrol-2-yl) porphyrin (**3a-2H**) S30

C) *meso* (*N*-Cbz-pyrrol-2-yl) porphyrin (**3b-2H**) S31

D) *meso* (*N*-Tos-pyrrol-2-yl) porphyrin (**3d-2H**) S32

E) *meso* (*NH*- pyrrol-2-yl) porphyrin (**3e-2H**) S35

7. MS spectra S37

1. General. Organic solutions were concentrated under reduced pressure on a Büchi rotary evaporator (< 37°C water bath). Chromatographic purification of products was carried out using Merck Kieselgel 60 silica gel (230-400 mesh). Thin-layer chromatography was carried out using Merck Kieselgel 60 F254 (230-400 mesh) aluminium plates with F-254 indicator and were visualized under UV light (254 nm, 320 nm). Solvent mixtures are understood as volume/volume. Spectroscopy: NMR (^{1}H- and ^{13}C-NMR) were recorded on a Varian AV300, AV400 or AV600 spectrometer (in ^{13}C: 75, 100 or 125 MHz) in CD_2Cl_2, or $CDCl_3$ and are reported relative to the solvents residual signal (^{1}H: C*HD*Cl$_2$, δ(H) 5.32 (t), C*H*Cl$_3$, δ(H) 7.26, ^{13}C: $\delta(^{13}CH_2Cl_2)$=54.0, $\delta(^{13}CHCl_3)$=77.2 ppm.). Data are reported in the following order: chemical shift (δ) in ppm; multiplicities are indicated s (singlet), bs (broad singlet), d (doublet), t (triplet), m (multiplet); coupling constants (*J*) are in Hertz (Hz), rounded to the nearest 0.1 Hz. UV-Vis spectra: *PerkinElmer Lambda 35 spectrometer*, in 10 mm quartz cells, λ_{max} (log ε) in nm. Fluorescence spectra: *PerkinElmer LS45* excitation (λ_{exc}) and emission wavelengths (λ_{Em} (intensity)) λ in nm. MS: ESI ThermoFisher LTQ-Orbitrap XL, positive ion mode, *m/z* (rel. intensity %) or a Finnigan SSQ 7000 mass spectrometer (EI or CI).

2. Experimental procedures for tetra (*N*-protected pyrrol-2-yl) porphyrin

A) $BF_3.OEt_2$ Method: In a dry flask, 1 mmol of N-(methane sulfonyl)-1H-pyrrol-2-aldehyde (4a, 1 eq.) and 100 mL abs CH_2Cl_2 were taken and purged the solution with argon, followed by addition of 70 µL (1 mmol, 69 mg, 1 eq.) of pyrrole. The resulting colourless solution was purged with argon for 5 min, and 0.2 mL of 0.5 M $BF_3.OEt_2$ (prepared by dissolving 0.5 mL of *ca.* 46% reagent in 7.5 mL CH_2Cl_2) solution (0.1 eq.) was added. This mixture was stirred for 1 h at r.t. under protection from light. Then, *p*-chloranil (1.5 mmol) was added and the mixture was refluxed for 1.5 h. The cold reaction was poured into sat $NaHCO_3$ (30 mL), extracted (3 x 30 mL) with CH_2Cl_2, dried over Na_2SO_4, and the solvents were removed on a rotary evaporator. Purification was carried out on silicagel 60 chromatography, using CH_2Cl_2/EtOAc gradient to obtain the products.

B) $NH_2OH{\cdot}HCl$: In a dry flask, 1 mmol of 4a (1 eq.) and 1 mmol (69.6 mg, 1 eq.) of hydroxylamine-hydrochloride ($NH_2OH{\cdot}HCl$) were taken, dried under H.V. for 2h, followed by addition of 5 mL abs chlorobenzene. The suspension was degassed for 10 min with argon bubbling, followed by the addition of 70 µL (1 mmol, 69 mg, 1 eq.) of pyrrole. The mixture was stirred for 24h at r.t. Then, nitrobenzene (3.75 mL) was added and the mixture was heated at 130°C for 2h. After cooling the reaction, solvents were removed under H.V. distillation. The residual black powder was washed with $NaHCO_3$ (30 mL) and extracted with (4 x 30 mL) CH_2Cl_2. Purification was carried out on silicagel 60 chromatography, using CH_2Cl_2/EtOAc gradient, and the isolated fractions were collected and the solvents removed on a rotary evaporator. The obtained brown product was washed with Et_2O, precipitated, and dried on HV.

C) $TiCl_4$: In a H.V. dry flask, 1 mmol of 4a (1 eq) and 70 µL (1 mmol, 69 mg, 1 eq.) of pyrrole were dissolved in 30 mL abs CH_2Cl_2, and the solution was purged with argon for 10 min. 220 µL

(2 mmol, 381 mg, 2 eq.) of $TiCl_4$ were added, and the resulting mixture was stirred at r.t. for 24h with frequent monitoring on TLC, and UV-vis-spectroscopy. The obtained brown slurry was poured in sat. $NaHCO_3$ (20 mL), and extracted with CH_2Cl_2 (4 x 30 mL), dried over Na_2SO_4, and solvents were removed on rotary evaporator. TLC analysis indicated no porphyrin characteristic product formation.

D) DMF/pTSA/heating: In a H.V. dry flask with Dean-Stark apparatus, 0.5 mmol 4a (1 eq) and 3.5 mL of abs DMF were mixed, and the resulting solution was degassed for 5 min with argon bubbling, followed by the addition of 35 µL (0.5 mmol, 34 mg, 1 eq) of pyrrole. The reaction mixture was heated in a pre-heated oil bath at 130°C for 10 min. To this hot solution, pTSA (90 mg, 0.5 mmol 1 eq.) in 1.5 mL of DMF was added, and the mixture was placed in a pre-heated oil bath at 150°C for 1 h. After cooling the reaction, the resulting brown mixture was poured in sat $NaHCO_3$ (20 mL), extracted with (4 x 20 mL) EtOAc, and organic extracts were washed with water (3x30 mL) and sat. NaCl solutions. The organic extracts were dried over Na_2SO_4 and solvents were removed on a rotary evaporator and H.V. removal of residual solvent. Purification was carried out on silicagel 60, using a CH_2Cl_2/EtOAc mixture and solvents were removed on rotary evaporator.

Metallation of (pyrrolyl) porphyrins

3c-Zn: In a round bottomed flask, 56.2 mg (0,064 mmol, 1 eq.) of *meso* tetra (1-(methylsulfonyl)-1H-pyrrol-2-yl) porphyrin taken and dissolved in 15 mL DMF. This brown suspension purged with argon for 5 min, and added 58.4 mg (0,318 mmol, 5 eq.) of $Zn(OAc)_2$ was added, reaction stirred in pre-heated oilbath at 100 °C for 1h. The obtained rose coloured solution poured in sat. $NaHCO_3$ (10 mL), extracted with EtOAc (4 x 15 mL) and organic extracts washed with water (3 x 20 mL), and dried on Na_2SO_4. The solvent was removed on rotary evaporator and obtained mixture filtered through a short silicagel 60 column, eluted with DCM. Upon removing solvents, the remaining violet solids dried on HV to obtain 59.8 mg (99 %) product.

3c-Ni: In a dry flask, 150 mg (0.17 mmol, 1 eq.) of *meso* tetra(1-(methylsulfonyl)-1H-pyrrol-2-yl) porphyrin in 15 mL DMF was taken. The solution purged with argon for 10 min, followed by added 540 mg (2.17 mmol, 12,8 eq.) $Ni(OAc)_2$ and solution stirred at 100 °C for 72 h with frequent monitoring of the progress. At the end, the solution poured into sat. $NaHCO_3$ solution (25 mL), extracted EtOAc (4 x 20 mL), organic extract washed with water (3 x 25 mL) and sat. NaCl, and dried over Na_2SO_4, removed on rotary evaporator. The obtained brown material filtered over a short silicagel column, using DCM/EtOAc (100:12). After removing the solvents on rotary evaporator, violet solids dried on HV to obtain 153 mg (96 %) of product.

Deprotection of tetra (Cby-pyrrol-2-yl) porphyrins (3e-2H)

In a round bottomed flask, 8.5 mg (7.87 µmol, 1 eq.) of *meso* tetra (1-carboxybenzyl)-1H-pyrrol-2-yl) porphyrin was taken in 4 mL THF and 1 mL of MeOH. The mixture purged with argon for 5 min, and added 1.5 mg 5% Pd/C. The reaction mixture stirred under hydrogen balloon atmosphere at room temperature for 60 h. The obtained green-coloured mixture was filtered through a 2 cm-cellite bed, washed with MeOH and removed all the solvents on rotary evaporator. The obtained mixture filtered through a 10 cm Alox (basic) column, eluted the product with 5% of MeOH in DCM. Upon removing solvents, the obtained green solid was dried on HV to obtain 4 mg (91 %) of product.

UV-vis and NMR confirm that product was protonated, which are clearly different than the reaction mixture UV-vis and NMR.

Table S1. Condensation of *N*-Me- or *N*-^{i}Pr-pyrrole-2-aldehyde and pyrrole

	Tetramerization Conditions[a]	*N*-Me Pyrrole 2-aldehyde	*N*-iPr Pyrrole 2-aldehyde
1	(a) 1 equiv. $NH_2OH.HCl$, chlorobenzene, r.t., 24 h;<br>(b) nitrobenzene, 130 °C, 2 h	Only starting materials;<br>n.r.	Only Starting materials<br>n.r.
2	(a) 0.1 to 1 equiv. BF_3OEt_2, CH_2Cl_2, r.t., 1 h;<br>(b) p-chloranil, 60 °C,1 h	Only starting materials;	Only starting materials;

[a] equimolar amounts of pyrrole and **4-R** (R = Me, ^{i}Pr) were used in the reaction. n.r.= neither product nor educts were found.

Table S2. Condensation of *N*-sulfonly-pyrrole-2-aldehyde (**4c** or **4d**) and pyrrole

No.	Tetramerization Conditions[a]	% yield (**3c-2H**) [b]	% yield (**3d-2H**) [b]
1	(a) DMF, 100 °C, 10 min;<br>(b) 1 equiv. pTSA, 150 °C, 1 h	< 5	22
2	2 equiv. $TiCl_4$, CH_2Cl_2, r.t., 1 h	n.r.	n.r.

[a] equimolar amounts of pyrrole and **4-R**, [b] combined yield of all atropisomers.

3. Analytical data for Compounds:

A) 3a-2H

Mixture of atropisomers; ^{1}H NMR (400 MHz, $CDCl_3$) δ (in ppm): 8.89-8.83 (8H, overlapped, porp-C*H*), 7.92 (4H, overlapped m, pyrrol-*H*), 7.16-7.03 (4H, overlapped m, pyrrol-*H*), 6.78 (4H, m, pyrrol-*H*), 6.6, 6.5, 6.4 (3xt, 4H), 6.19 (4H, m), 6.04 (3H, br t), 5.96-5.86 (5H, overlapped m), 5.74 (d) overlapped with 5.68 (1H, t), 5.44 (m, 2H), 4.45-4.10 (overlapped d, PhCH_2-O), -2.7 (br s), -2.87 (br s), - 2.90 (br s, *NH*).

ESI MS (calc. $C_{56}H_{59}N_8O_8^+$: 971.44504), m/z_{found} = 971.44543 (100%, $[M+H]^+$)

B) 3b-2H

Mixture of atropisomers; ^{1}H NMR (400 MHz, $CDCl_3$) δ (in ppm): 8.9-8.7 (8H, overlapped, porp-C*H*), 7.4-7.1 (20H, overlapped m, Ph-*H*), 6.78 (4H, m), 7.02 (m), 6.6 – 5.9 (overlapped m), 5.74 (1H, dd, *J* = 1.5, 3.1 pyrrol-*H*), 6.72-6.66 (overlapped m, pyrrol-*H*), 0.15 (s), 0.1 (s, tBu); 0.0 (s, 9H, tBu), -0.04 (s, 9H, tBu), -0.18 (s,), -0.26 (s, tBu),

ESI MS (calc. $C_{68}H_{51}N_8O_8^+$: 1107.38244), m/z_{found} = 1107.38245 (100%, $[M+H]^+$)

C) Individual atropisomers of 3c-2H

F1: ^{1}H NMR (600 MHz, CD_2Cl_2) δ (in ppm): − 2.73 (2H, s, N*H*), 2.59 (12H, s, C*H*3), 6.92 (4H, t, *J* = 3.45 Hz, pyrrol-*H*), 7.46 (4H, dd, *J* = 1.78, 3.18 pyrrol-*H*), 7.75 (4H, dd, *J* = 1.76, 3.62 Hz, pyrrol-*H*), 8.99 (8H, s, fwhm 6 Hz, porp-C*H*). ^{13}C NMR (150 MHz, CD_2Cl_2) δ (in ppm): 132.7, 123.8, 122.5, 111.8, 109.1, 43.4. (fwhm: full width at half maximum)

HR MS: calc $C_{40}H_{35}N_8O_8S_4^+$: 883.14552, m/z_{found} = 883.14557 (100%), (Δm = 0.00005 Da)

ESI MS (calc $C_{40}H_{35}N_8O_8S_4^+$: 883.14552), m/z_{found} = 883.14471 (100%, $[M+H]^+$)

F2: ^{1}H NMR (600 MHz, CD_2Cl_2) δ (in ppm): −2.81 (2H, s, N*H*), 2.68 (12H, s, C*H*3), 6.89 (4H, t, *J* = 3.4 Hz, pyrrol-*H*), 7.31 (4H, dd, *J* = 1.79, 3.16 Hz, pyrrol-*H*), 7.79 (4H, dd, *J* = 1.76, 3.63 Hz, pyrrol-*H*), 8.91 (4H, *s*, porp-C*H*), 9.02 (4H, *s*, C*H*).

ESI MS (calc. $C_{40}H_{35}N_8O_8S_4^+$: 883.14552), m/z_{found} = 883.14539 (76%, $[M+H]^+$)

F3: ^{1}H NMR (600 MHz, CD_2Cl_2) δ (in ppm) :− 2.79 (2H, s, N*H*), 2.58 (6H, s, C*H*3), 2.67 (3H, s, C*H*3), 2.71 (3H, s, C*H*3), 6.87 (1H, t, *J* = 3.44 Hz, pyrrol-*H*), 6.90 (2H, t, *J* = 3.47 Hz, pyrrol-*H*), 6.91 (1H, t, *J* = 3.45 Hz, pyrrol-*H*), 7.23 (1H, dd, *J* = 1.79, 3.18 Hz, pyrrol-*H*), 7.38 (2H, dd, *J* = 1.79, 3.19, pyrrol-*H*), 7.39 (1H, dd, *J* = 1.79, 3.18 Hz, pyrrol-*H*), 7.77 (3H, dd, *J* = 1.82, 3.65 Hz, pyrrol-*H*), 7.80 (1H, dd, *J* = 1.76, 3.63 Hz, pyrrol-*H*), 8.9-9.05 (8H, porp-C*H*).

^{13}C NMR (150 MHz, CD_2Cl_2) δ = 132.9, 132.8, 124.1, 123.9, 123.8, 122.7, 122.2, 122.0, 111.9, 111.5, 111.4, 109.3, 109.1, 43.47, 43.30.

ESI MS (calc $C_{40}H_{35}N_8O_8S_4^+$: 883.14552), m/z_{found} = 883.14404 (100%, $[M+H]^+$)

F4: ^{1}H NMR (600 MHz, CD_2Cl_2) δ (in ppm): − 2.77 (2H, s, NH), 2.51 (12H, s, C*H3*), 6.88 (4H, t, J = 3.43 Hz, pyrrol-H), 7.30 (4H, dd, J = 1.78, 3.08 Hz, pyrrol-H), 7.79 (4H, dd, J = 1.68, 3.55 Hz, pyrrol-H), 8.95 (8H, br s, fwhm: 12 Hz, porp-*C*H). (fwhm: full width at half maximum)

^{13}C NMR (150 MHz, CD2Cl2) δ (in ppm): 132.9, 124.1, 121.8, 111.3, 108.9, 43.2.

ESI MS (calc $C_{40}H_{35}N_8O_8S_4^+$: 883.14552), m/z_{found} = 883.14459 (100%, $[M+H]^+$)

D) 3d-2H

Atropisomer minor fraction, F1: ^{1}H NMR (400 MHz, $CDCl_3$) δ (in ppm): -2.74 (2H, br s, NH), 2.45 (12H, s, Tos-CH_3), 6.76 (8H, d, J =3.5 Hz, pyr-H), 6.85 (8H,m, pyr-H), 6.99 (8H, d, J =6.9 Hz, Tos-H), 7.14 (8H, d, J =6.9 Hz, Tos-H), 7.97 (8H, m, pyr-H),8.33 (8H, s, porp-H),

^{13}C NMR (150 MHz, $CDCl_3$) δ (in ppm): 145.4, 135.1, 132.9, 130.1, 127.3, 123.2, 122.1, 111.4, 108.7, 29.4, 21.6.

ESI (calc $C_{64}H_{51}N_8O_8S_4^+$: 1187.27072), found: 1187.27271 (35%, $[M+H]^+$); 1225.22791 [M+K]+

Mixture of non-separable atropisomer fraction, F2: ^{1}H NMR (400 MHz, $CDCl_3$) δ (in ppm): -2.91, -2.85 (2H, br s, N*H*), 2.06, 2.15, 2.27, 2.41, (12H, 4 s, C*H3*), 6.11-6.37 (4H, m, pyrrol-*H*), 6.77-6.97 (8H, m, Ar-*H*), 7.10-7.32 (8H, m, Ar-*H*), 7.94-8.00 (4H, m, pyrrol-*H*), 8.15-8.53 (8H, m, porp-*H*).

ESI (calc $C_{64}H_{53}N_8O_8S_4^+$: 1189.2863), found: 1189.2851 ($[M+H]^+$)

3e-2H: ^{1}H NMR (600 MHz, CD_3OD) δ (in ppm): 8.45 (s), 7.87 (br m, J < 1.5 Hz, pyrrol-H), 7.58 (m, J = *ca*, 2.1, 4.1 Hz, pyrrol-H), 6.97 (m, J = 7.3, pyrrol-H).

$[3e\text{-}2H+H_n]^+$: ^{1}H NMR (600 MHz, CD_3OD) δ (in ppm): 6.8 (s), 3.75 (t, J = 7 Hz, pyrrol-H), 3.2 (q, J = 7.2 Hz), 2.57 (t, J = 7.3, pyrrol-H), 2.11 (1H, s), 1.30 (t, J = 7.4) overlapped with 1.28 (s).

ESI MS (calc $C_{36}H_{27}N_8^+$: 571.23532), m/z_{found} = 571.23407 ($[M+H]^+$)

3c-Zn: ^{1}H-NMR (400 MHz, $CDCl_3$): δ (in ppm): 2.49-2.58 (m, 12H, C*H3*), 6.80-6.90 (m, 4H, pyrrol-*H*), 7.29-7.40 (m, 4H, pyrrol-*H*), 7.65-7.78 (m, 4H, pyrrol-*H*), 8.99 (m, 8H, porp-*H*).

3c-Ni: ^{1}H-NMR (400 MHz, $CDCl_3$): δ (in ppm): 2.39-2.61 (s, 12H, C*H3*), 6.69-6.83 (m, 4H, pyrrol-*H*), 7.18-7.65 (m, 8H, pyrrol-*H*), 8.70-8.92 (m, 8H, porp-*H*).

4. UV-Vis and Fluorescence Spectra

A) 3c-2H atropisomers

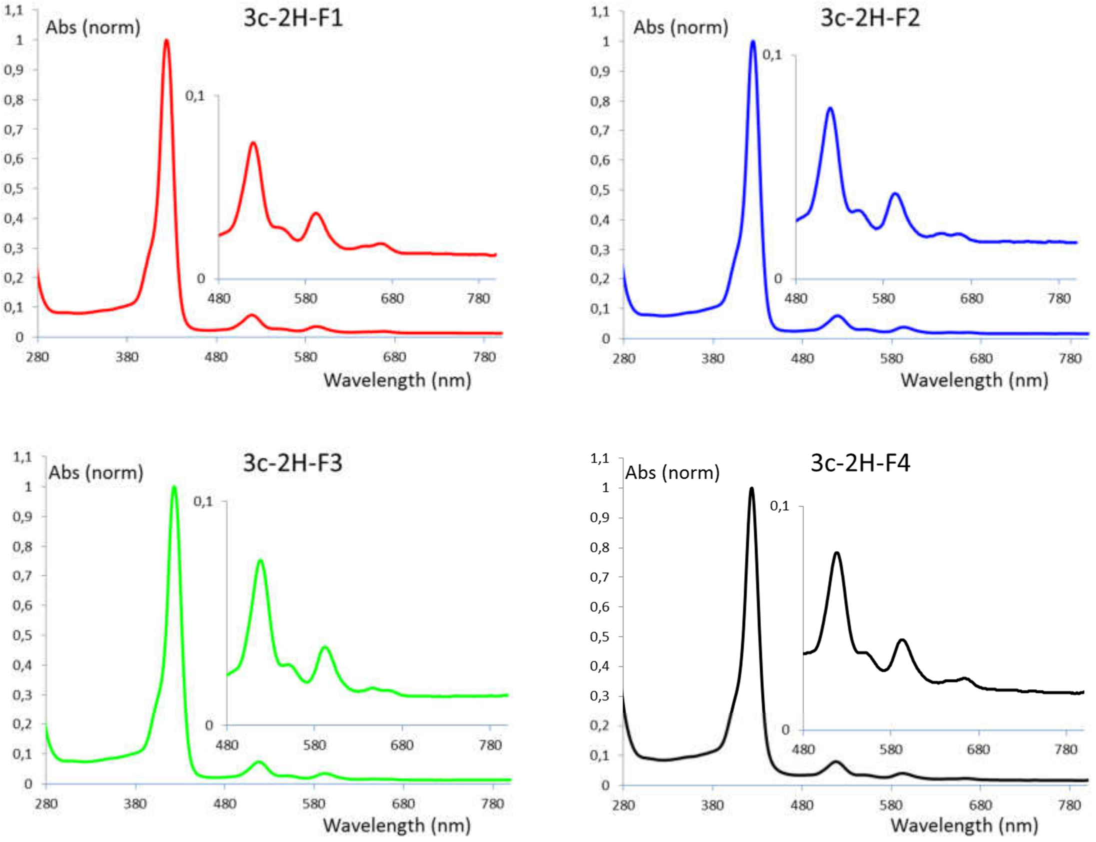

Fig S1: Normalized UV-Vis (in $CHCl_3$) of *meso* (*N*-Ms-pyrrol-2-yl) porphyrin (**3c-2H**).

B) Fluorescence Emission and Excitation Spectra of 3c-2H

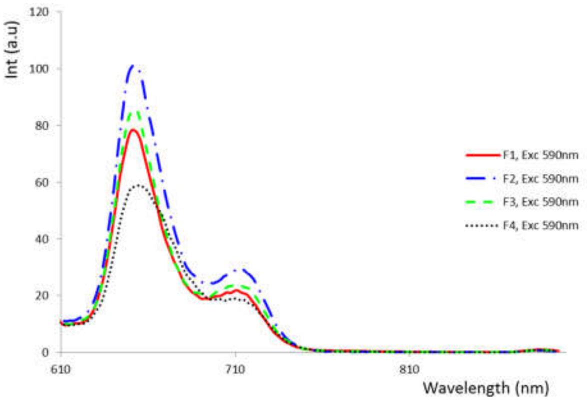

Fig S2: Fluorescence emission spectra (for λ_{exc} at 590 nm, in CH_2Cl_2) of **3c-2H** atropisomer fractions.

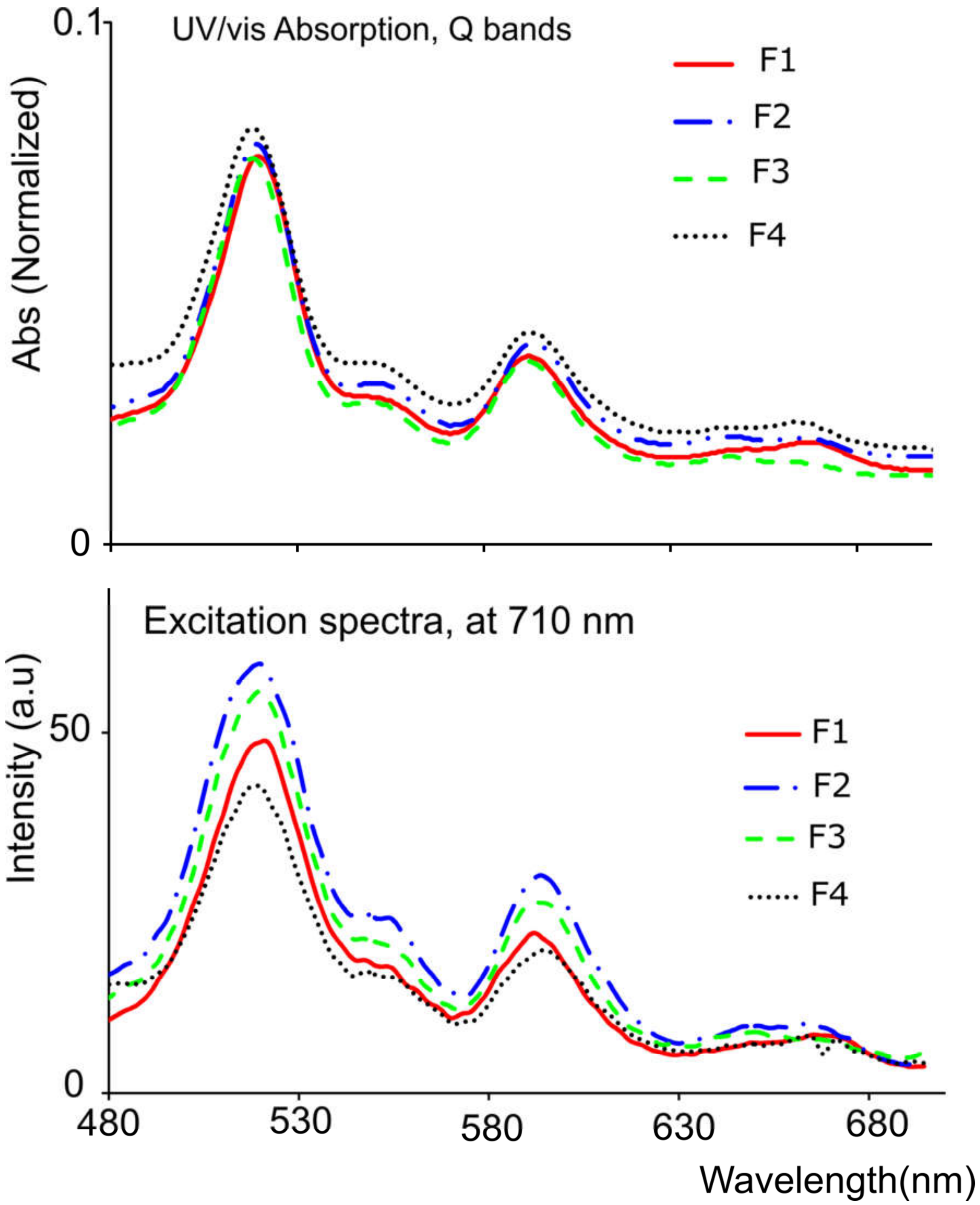

Fig S3: Top: UV-Vis of Q band region, Bottom: Excitation spectra (for λ_{emt} at 710 nm) of **3c-2H** atropisomers (in CH_2Cl_2).

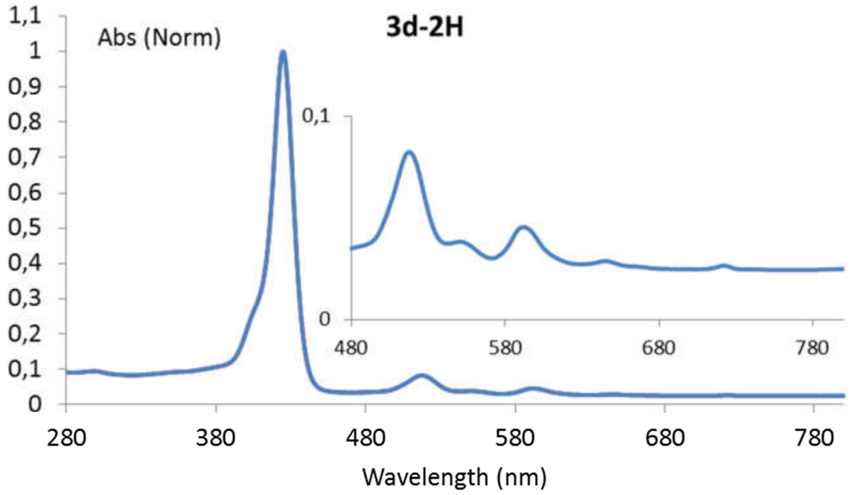

Fig S4: Normalized UV-Vis-absorption of *meso* tetra (*N*-tosyl pyrrol-2-yl) porphyrin (F1, **3d-2H** in CH_2Cl_2)

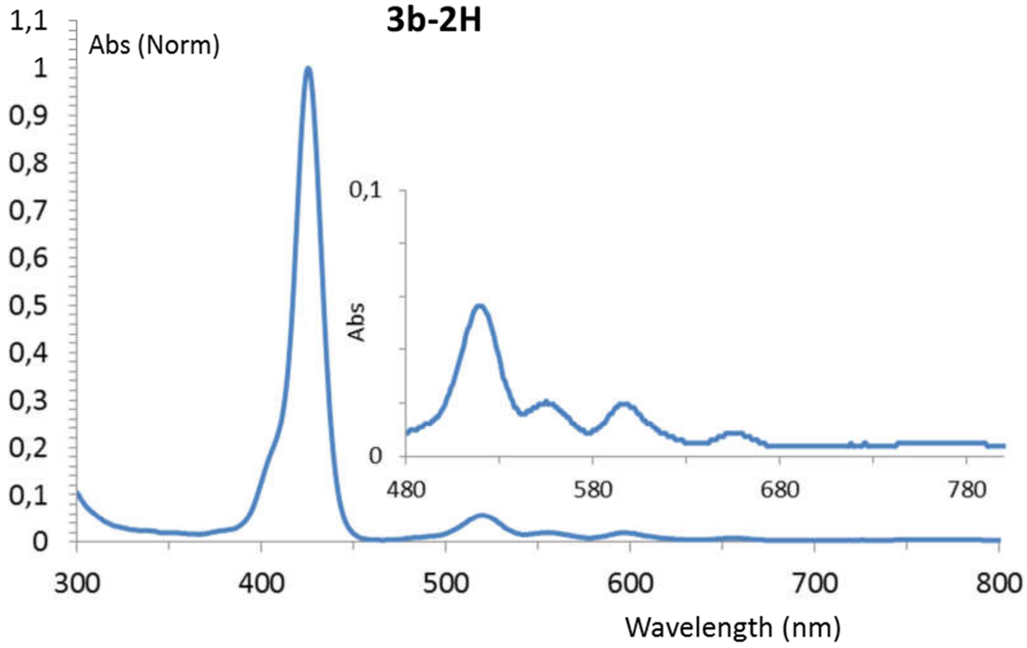

Fig S5: Normalized UV-Vis-absorption of *meso* tetra (*N*-Cbz-pyrrol-2-yl) porphyrin (**3b-2H**, CH_2Cl_2)

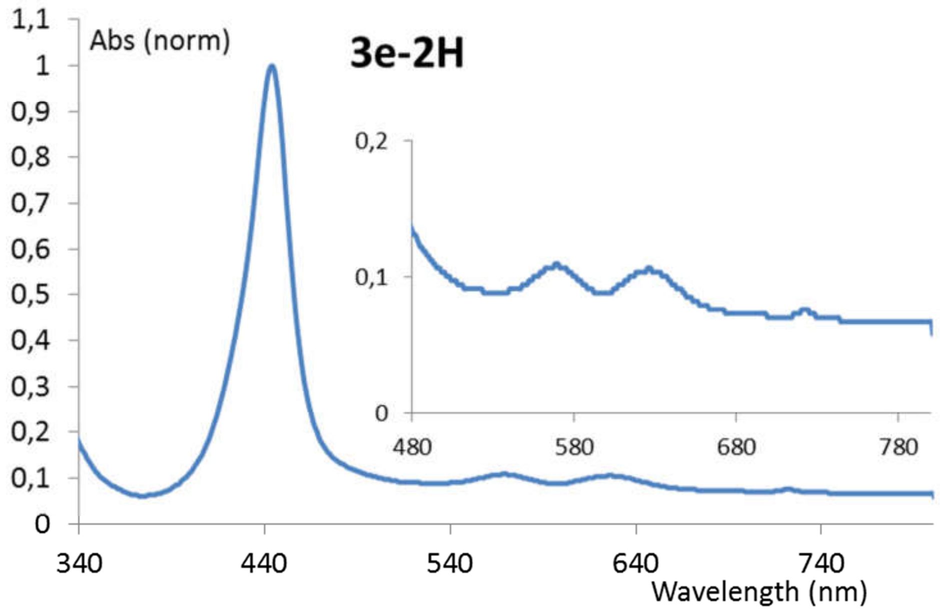

Fig S6: Normalized UV-Vis-absorption spectra of *meso* tetra (*NH*-pyrrol-2-yl) porphyrin **3e-2H** (in CH_2Cl_2)

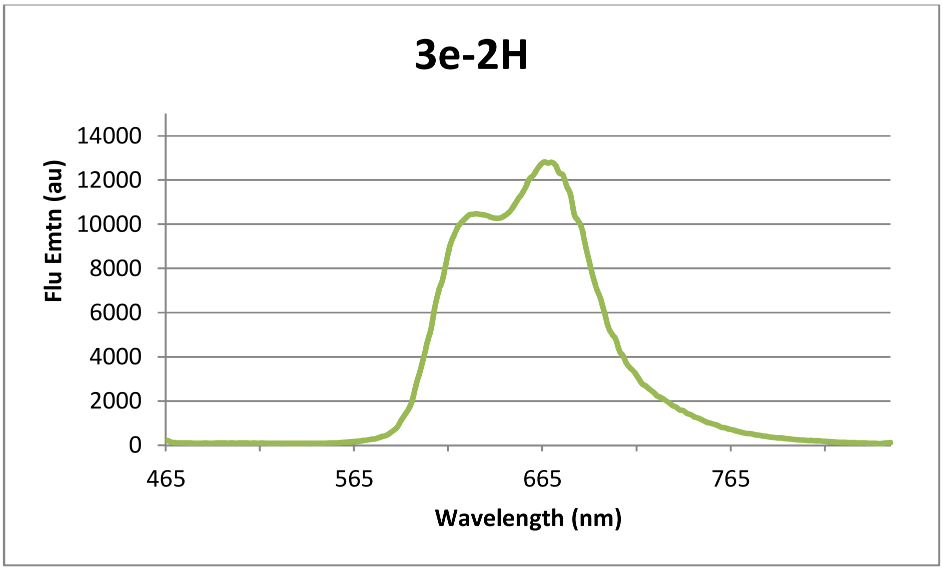

Fig S7: Fluorescence emission of *meso* tetra (*NH* pyrrol-2-yl) porphyrin **3e-2H** (for λ_{Exc}: 434 nm, in DMSO).

5. HPLC Chromatography

Flow rate 1mL/min, Kromasil Si-100 (unmodified silica, 7µM particle size), 250x4.6mm column

Gradient 1: The TOC image (HPLC traces of **3c-2H** atropisomers) data was obtained using the following isocratic gradient; *$CHCl_3$:EtOH, 85:15, for 60 min;*

Gradient 2 (for 3a-2H, and 3b-2H): *$CHCl_3$: EtOAc gradient (0 to 20 min, 100:0; 20 to 30 min, 98:2; 30 to 40 min, 90:10; 40 to 55 min, 85:15; 55 to 65 min, 85:15; 65 to 75, 100:0, 75 to 90 min, 100:0)*.

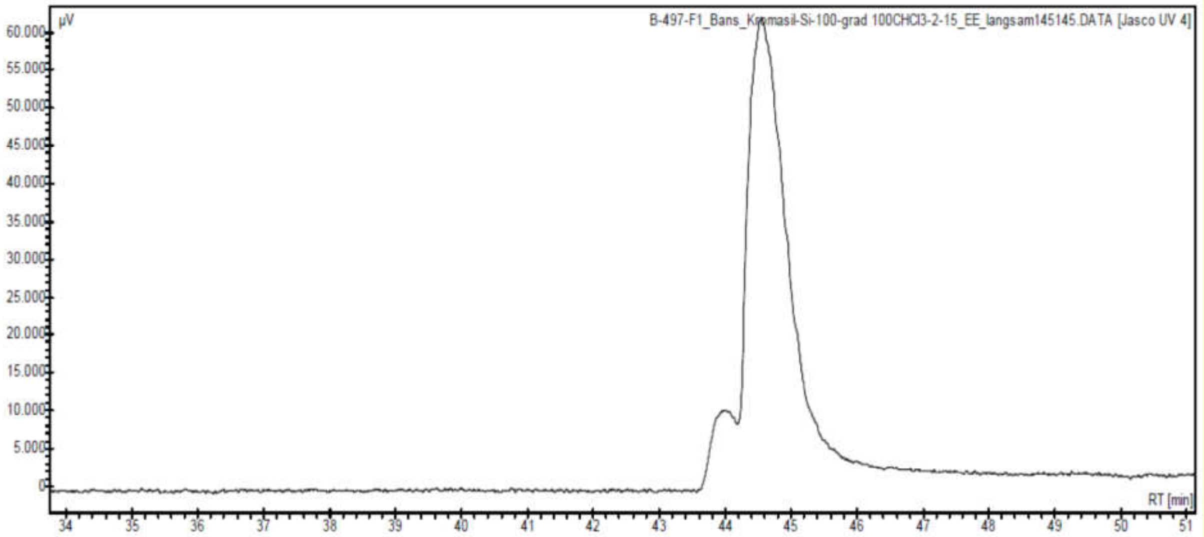

Fig S8: HPLC chromatogram of *meso* tetra (*N*-Boc-pyrrol-2-yl) porphyrin (**3a-2H,** trace detection at 400 nm)

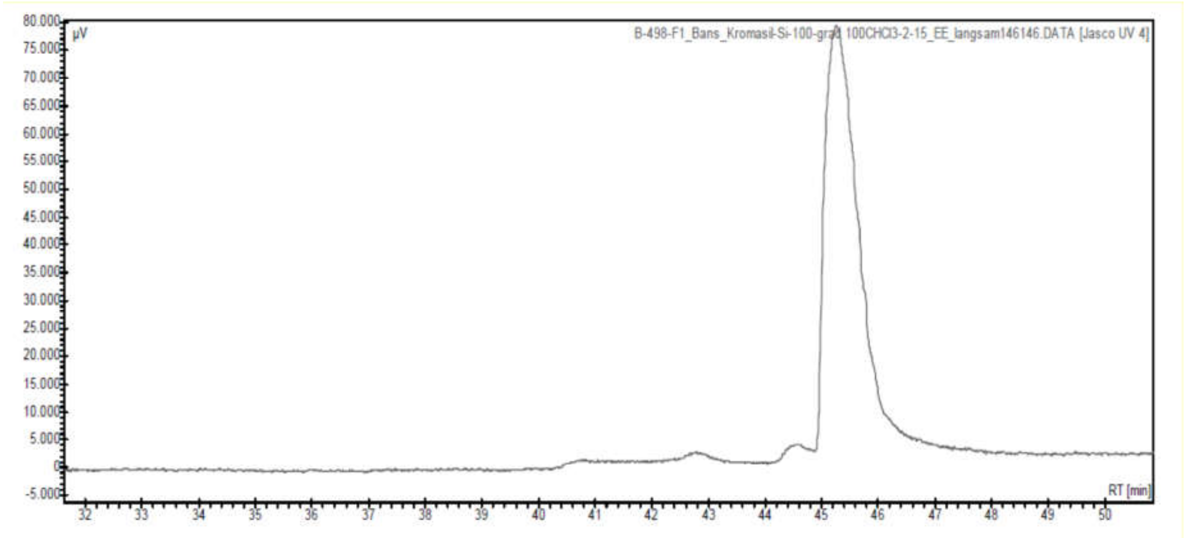

Fig S9: HPLC chromatogram of *meso* tetra (*N*-Cbz-pyrrol-2-yl) porphyrin (**3b-2H**, detection at 400 nm)

Modified Gradient 3 (for 3c-2H):

$CHCl_3$: EtOH gradient (0 to 20 min, 100:0; 20 to 30 min, 98:2; 30 to 40 min, 90:10; 40 to 55 min, 85:15; 55 to 65 min, 85:15; 65 to 75, 100:0, 75 to 90 min, 100:0);

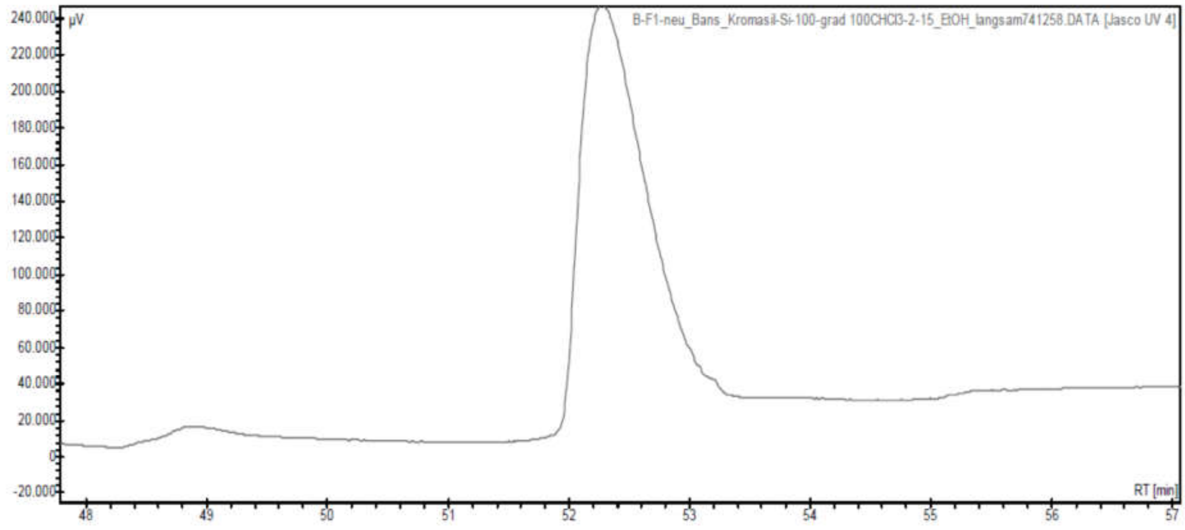

Fig S10: HPLC chromatogram of **F1** of *meso* tetra (*N*-Ms-pyrrol-2-yl) porphyrin of **3c-2H** (trace at 400 nm)

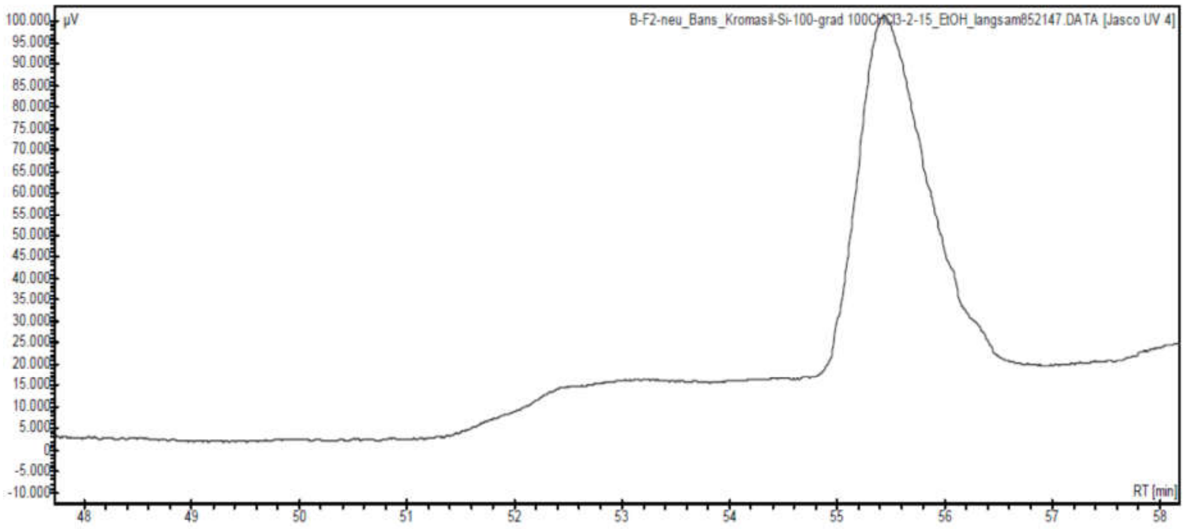

Fig S11: HPLC chromatogram of **F2** of *meso* tetra (*N*-Ms-pyrrol-2-yl) porphyrin **3c-2H** (trace at 400 nm)

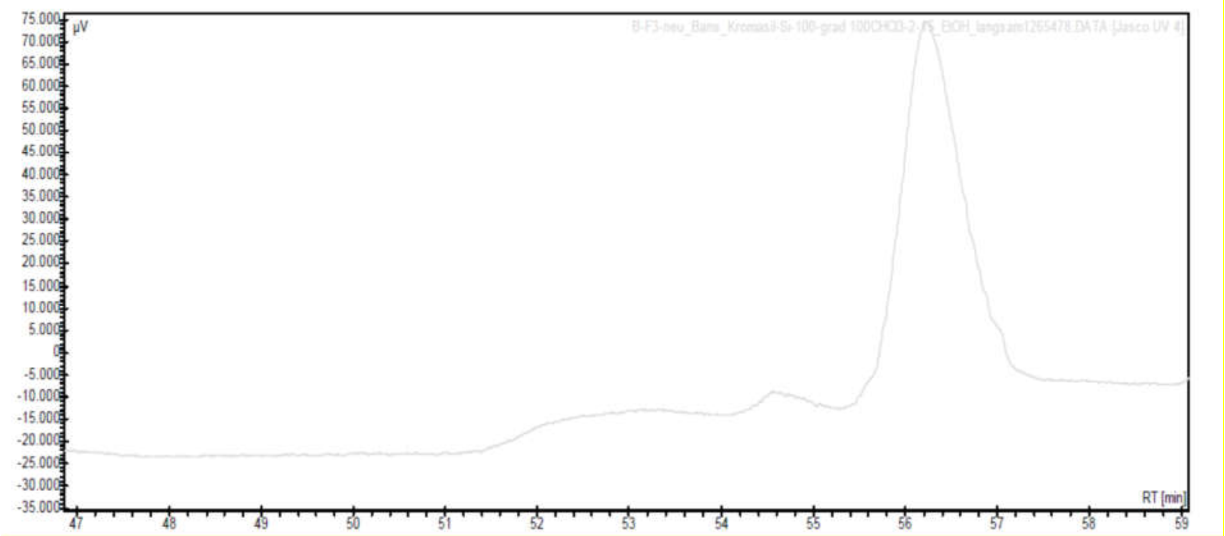

Fig S12: HPLC chromatogram of **F3** of *meso* tetra (*N*-Ms-pyrrol-2-yl) porphyrin **3c-2H** (trace at 400 nm)

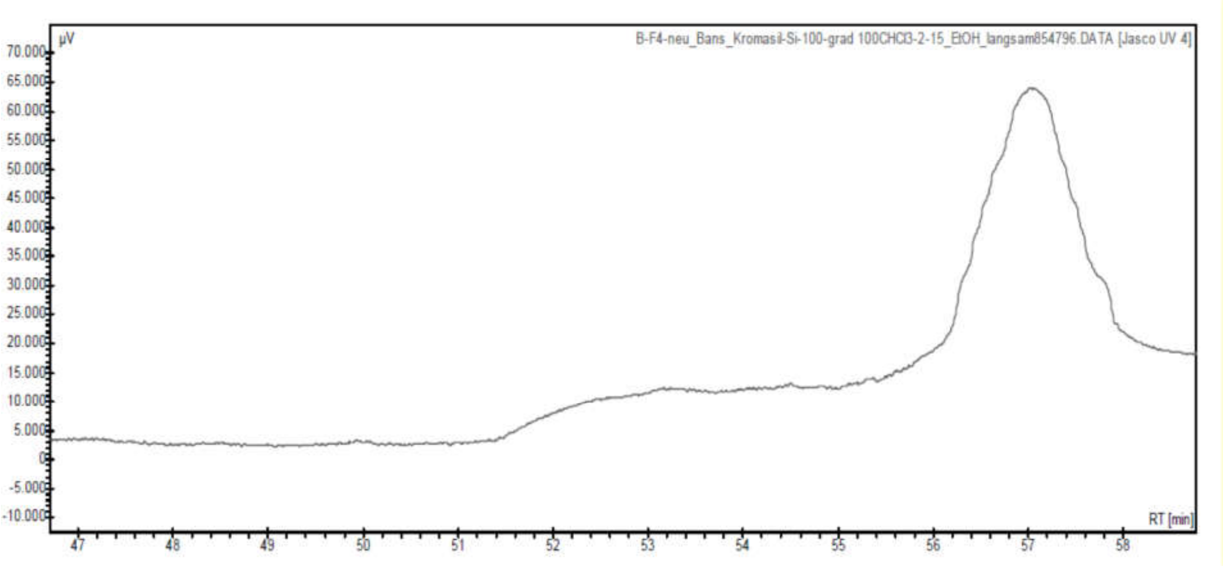

Fig S13: HPLC chromatogram of **F4** of *meso* tetra (*N*-Ms-pyrrol-2-yl) porphyrin **3c-2H** (trace at 400 nm)

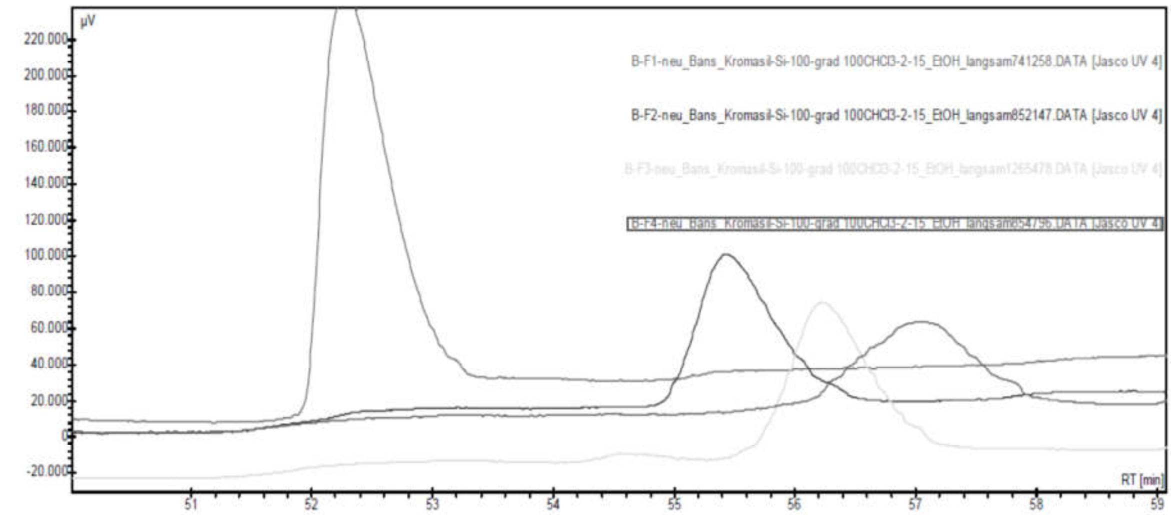

Fig S14: Overlay of HPLC chromatograms of *meso* tetra (*N*-Ms-pyrrol-2-yl) porphyrin **3c-2H** atropisomers (traces at 400 nm in gradient 3).

After heating 3c-2H-F3 at 100 °C for 4h (the V T NMR experiment for isomerization):

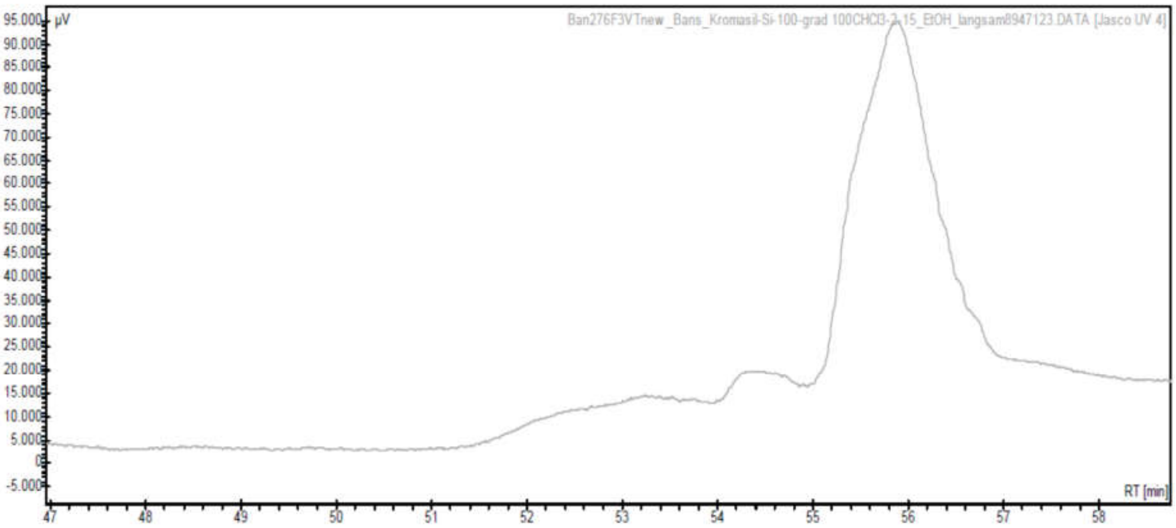

Fig S15: HPLC chromatogram of **3c-2H-F3 after heating for 4h at 100 °C** (trace at 400 nm)

Overlay with other isomers 3c-2H, and F3 after heating at 100 °C for 4h,

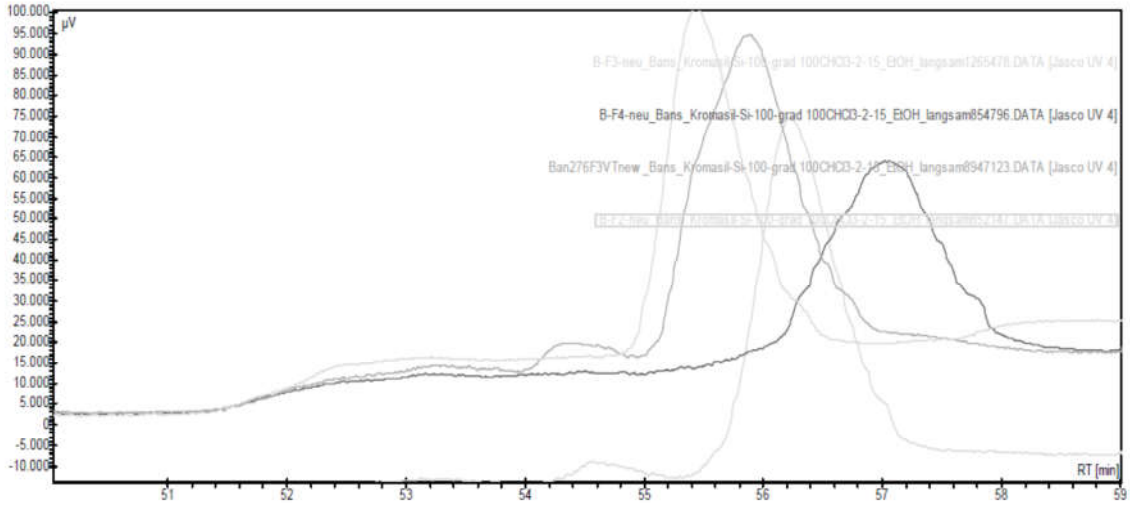

Fig S16-1: Overlay of chromatograms with other isomers (400 nm detection) **3c-2H-F3 after heating for 4h at 100 °C** (HPLC system generated image) and bottom chromatogram as colour image)

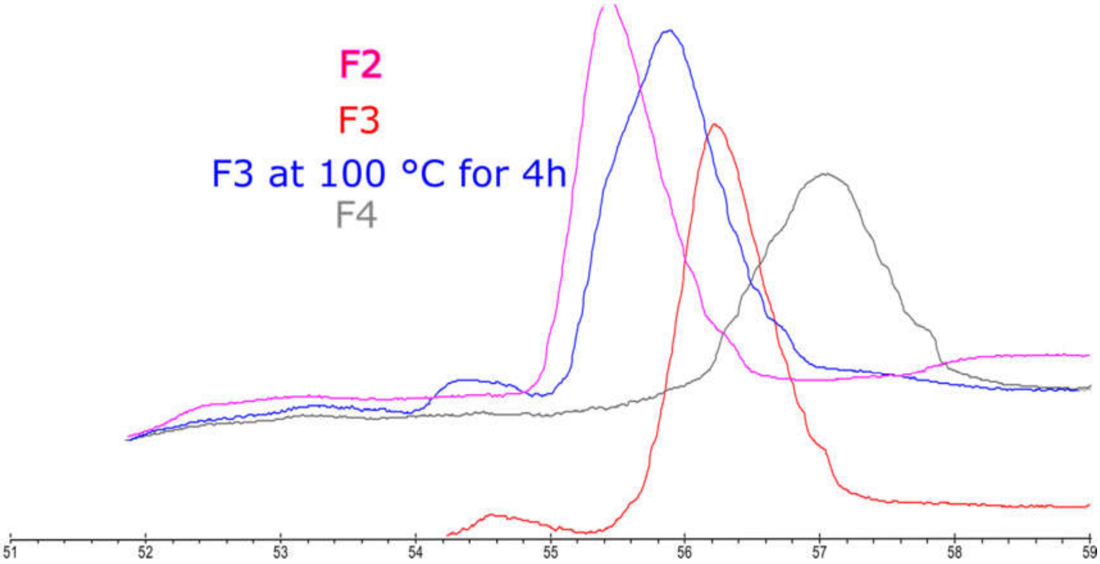

Fig S16-2: Overlay of chromatograms **3c-2H-F3 after heating for 4h at 100 °C** with other isomers (400 nm detection)

6. NMR Spectra:

A) *meso* (*N*-mesyl pyrrol-2-yl) porphyrin (3c-2H)

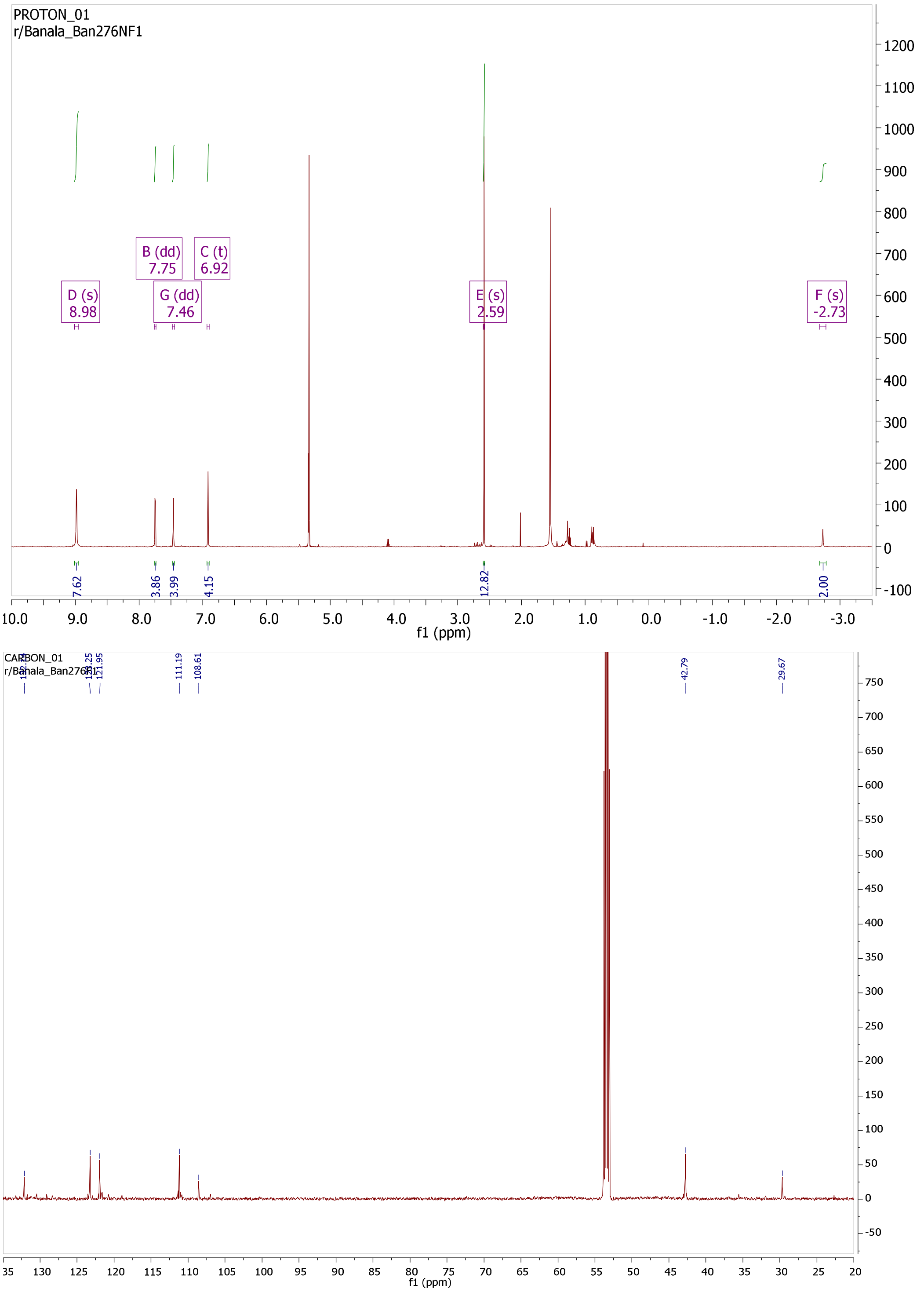

Fig S17: ^{1}H and ^{13}C NMR (600 MHz, 125 MHz in CD_2Cl_2) of apolar fraction F1 of **3c-2H**

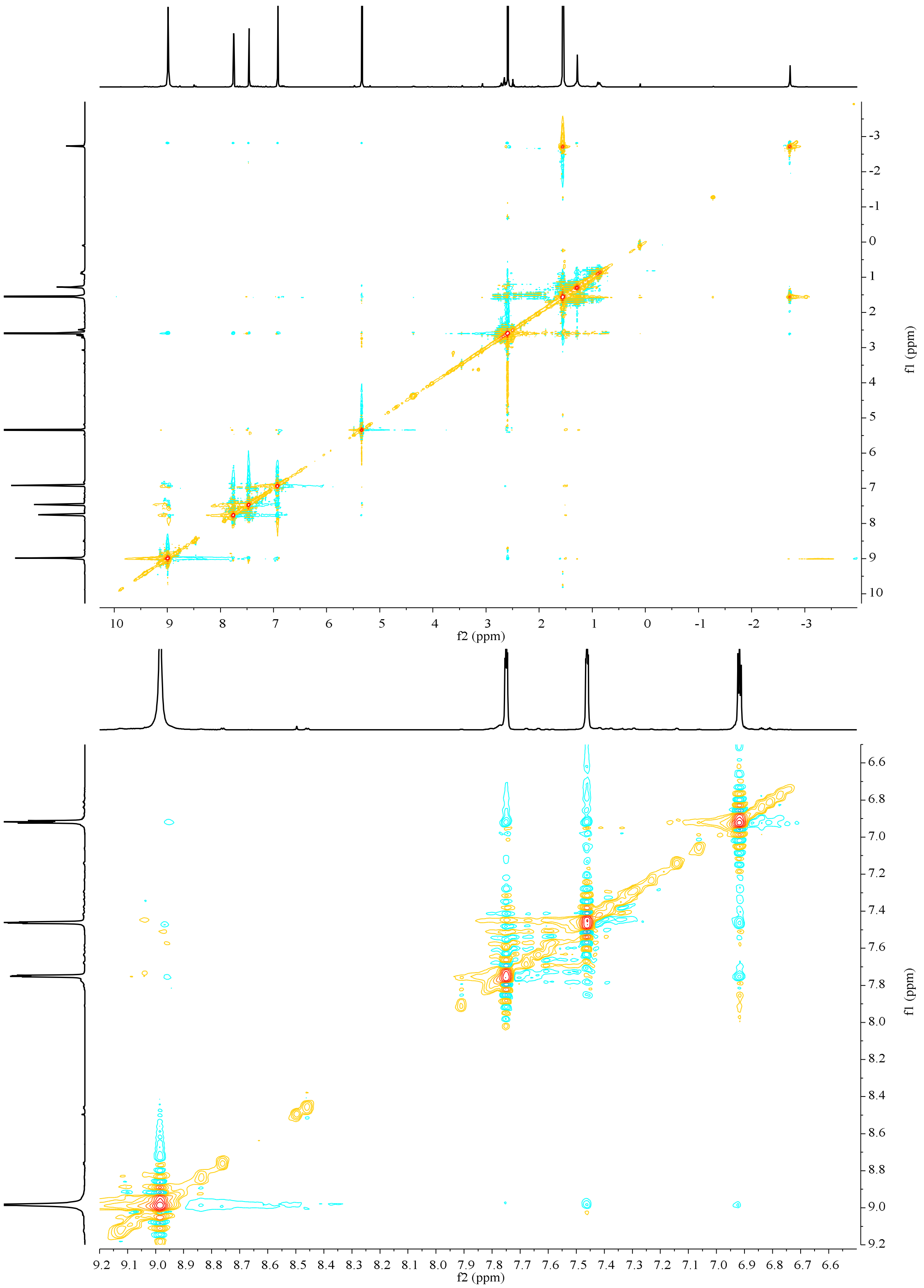

Fig S18: NOESY spectrum (in CD_2Cl_2, 600MHz) of apolar fraction **F1** of **3c-2H**, bottom expanded aromatic region.

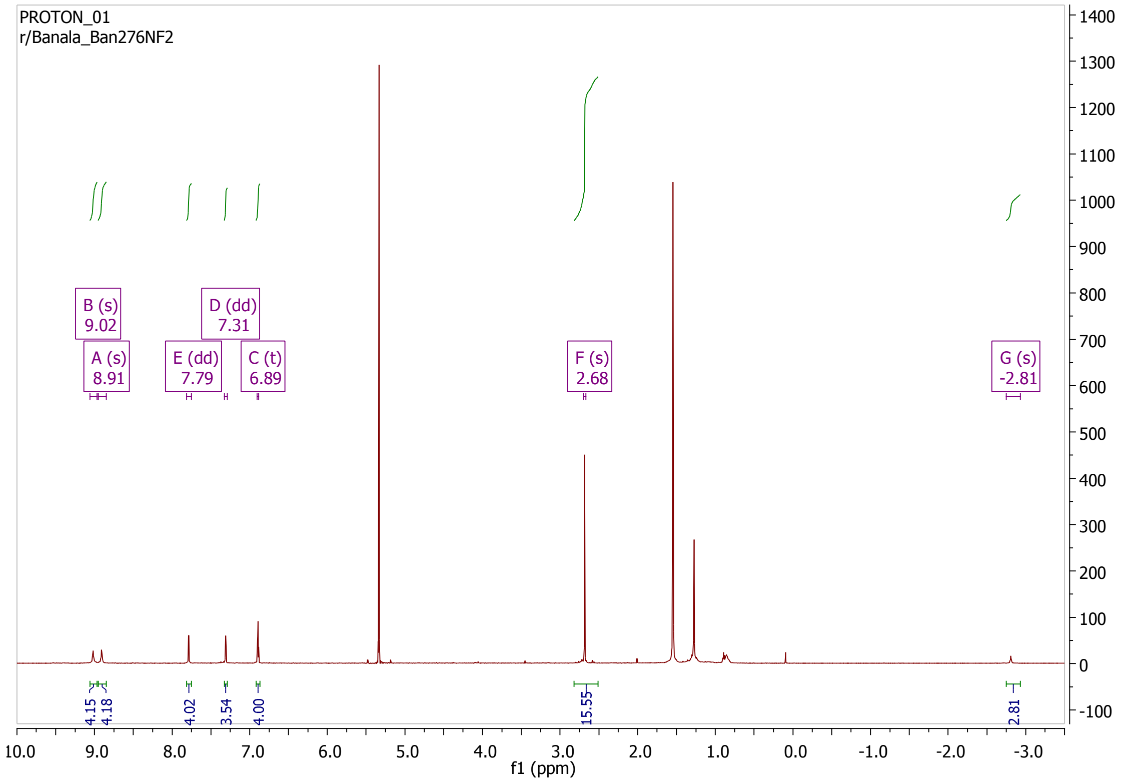

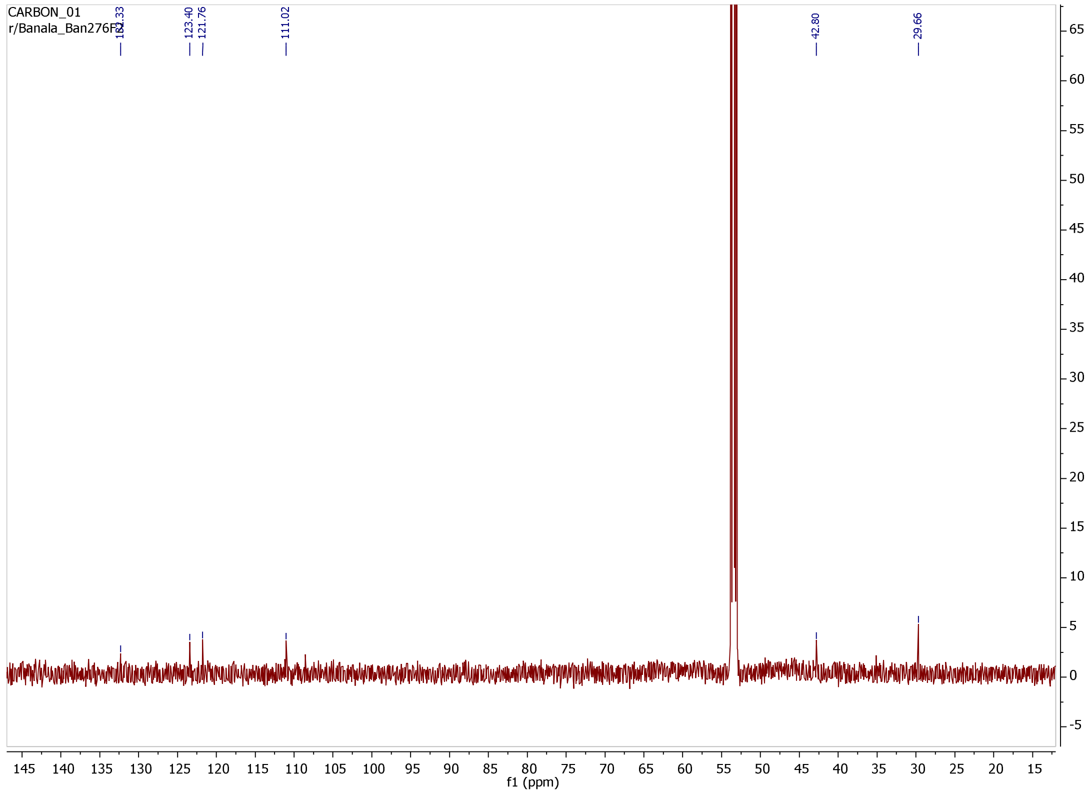

Fig S19: ^{1}H, ^{13}C NMR (600 MHz, 125 MHz in CD_2Cl_2) of medium polar fraction F2 of **3c-2H**

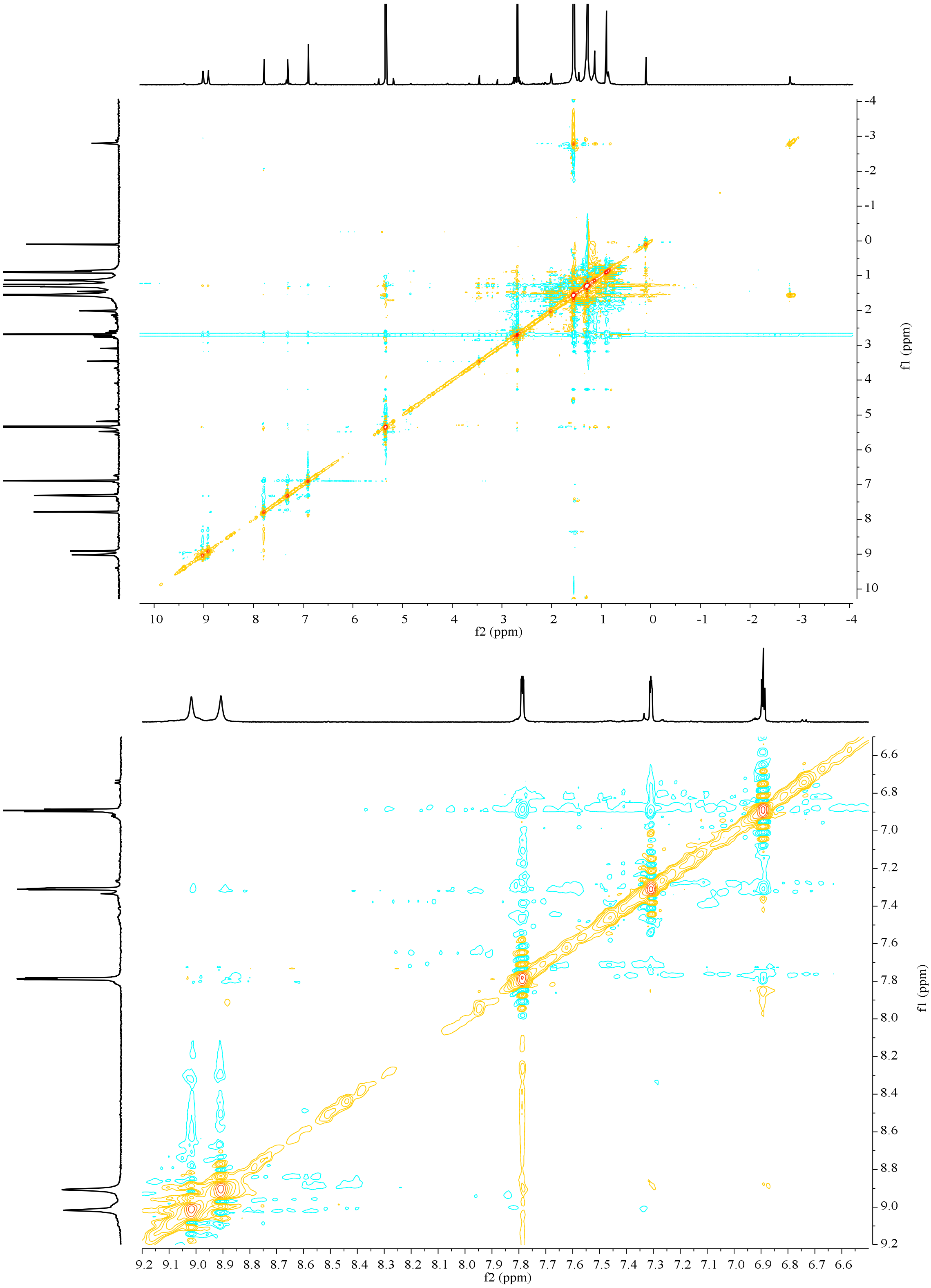

Fig S20: NOESY spectrum (in CD_2Cl_2, 600MHz) of medium polar fraction **F2** of **3c-2H**, bottom expanded aromatic region.

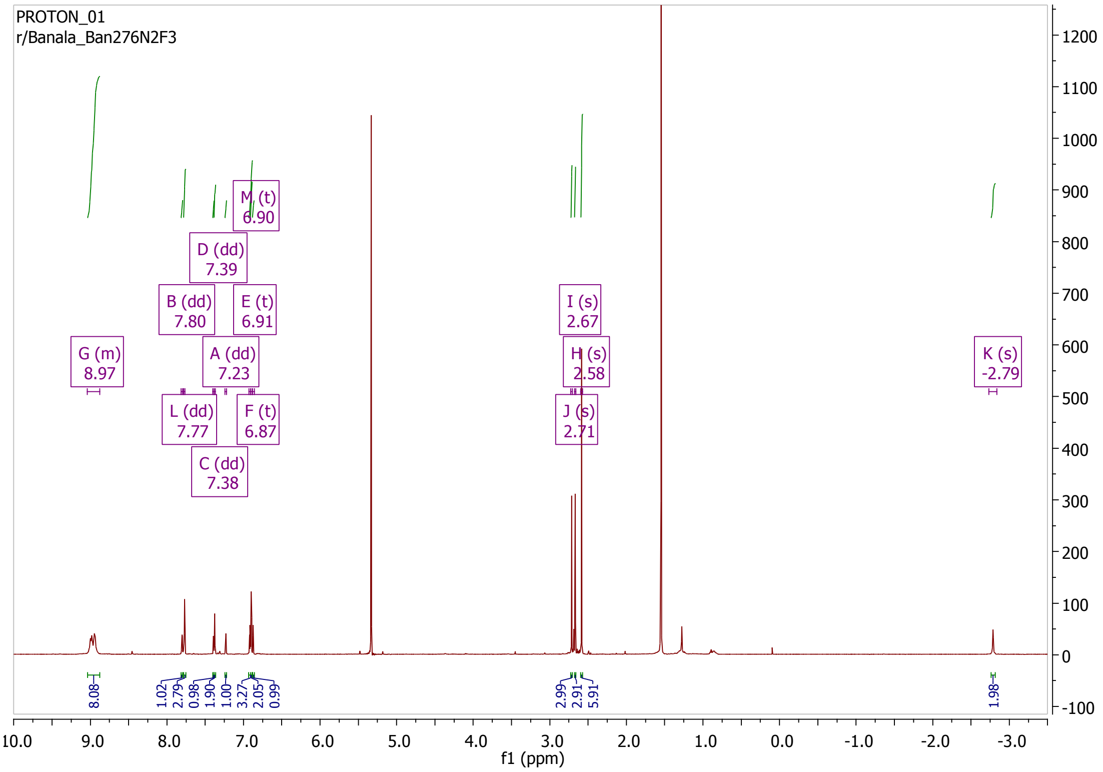

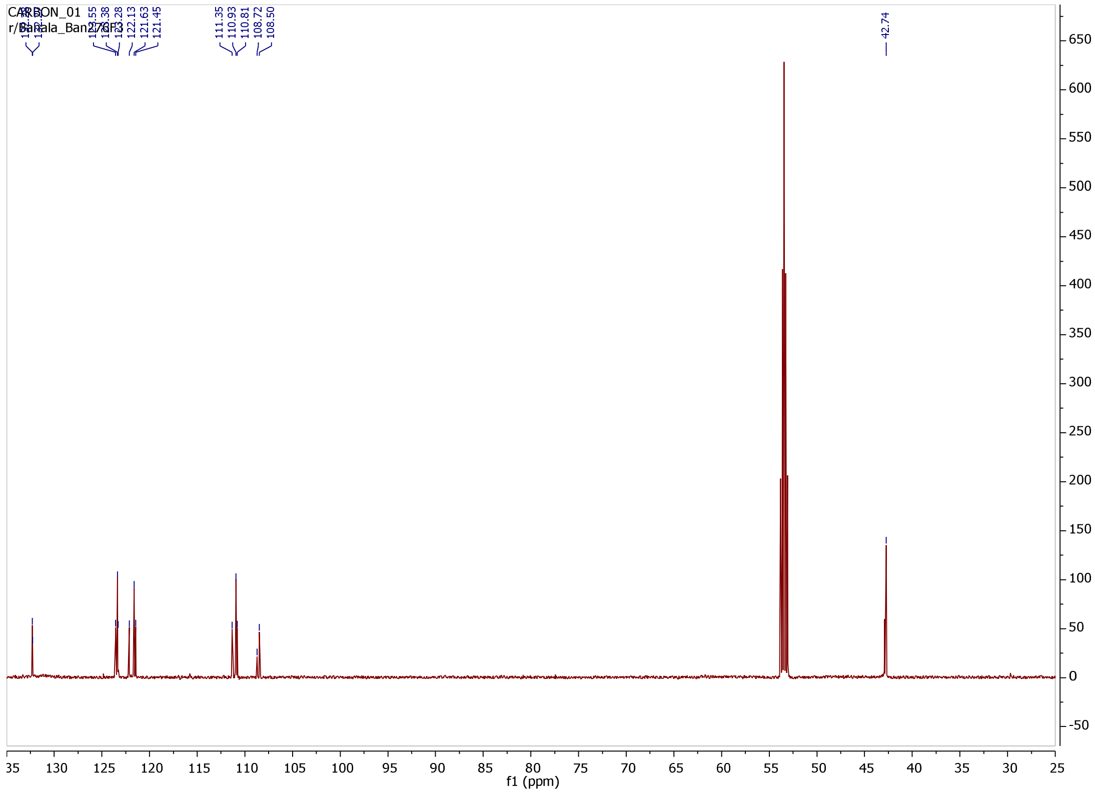

Fig S21: ^{1}H and ^{13}C NMR (600, 125 MHz, CD_2Cl_2) of medium polar fraction F3 of **3c-2H**

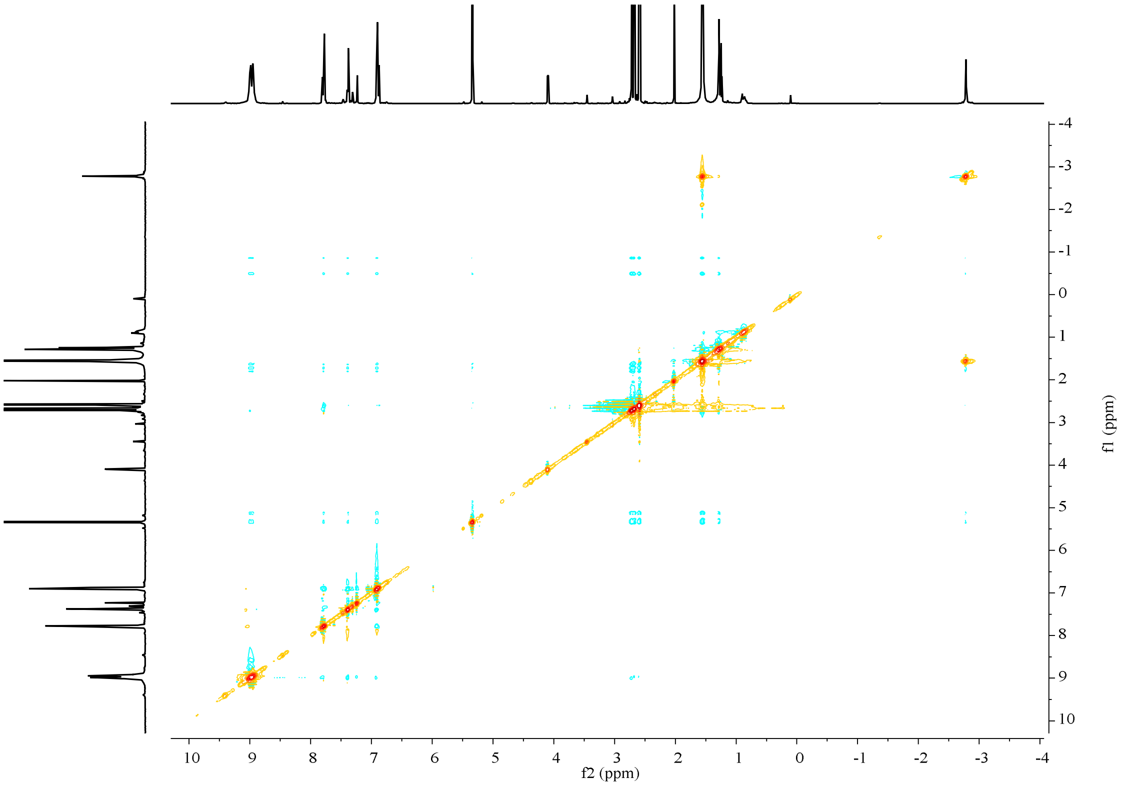

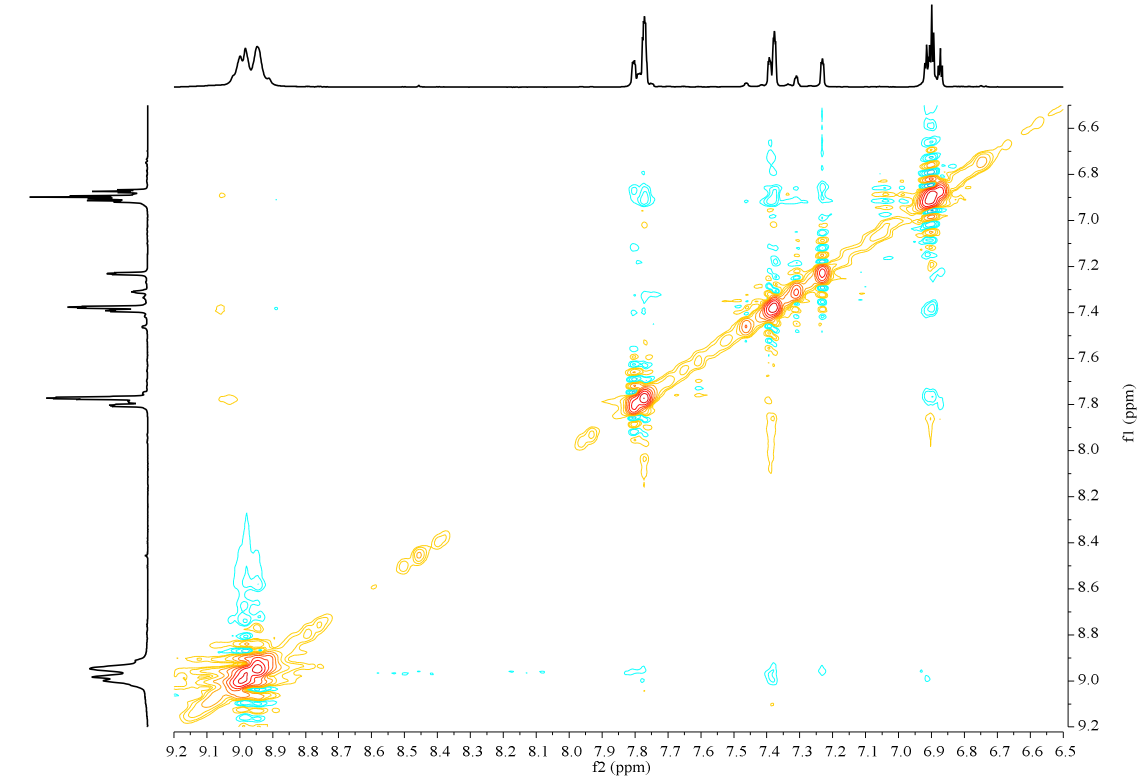

Fig S22: NOESY spectrum (in CD_2Cl_2, 600MHz) of medium polar fraction **F3** of **3c-2H**, bottom expanded aromatic region.

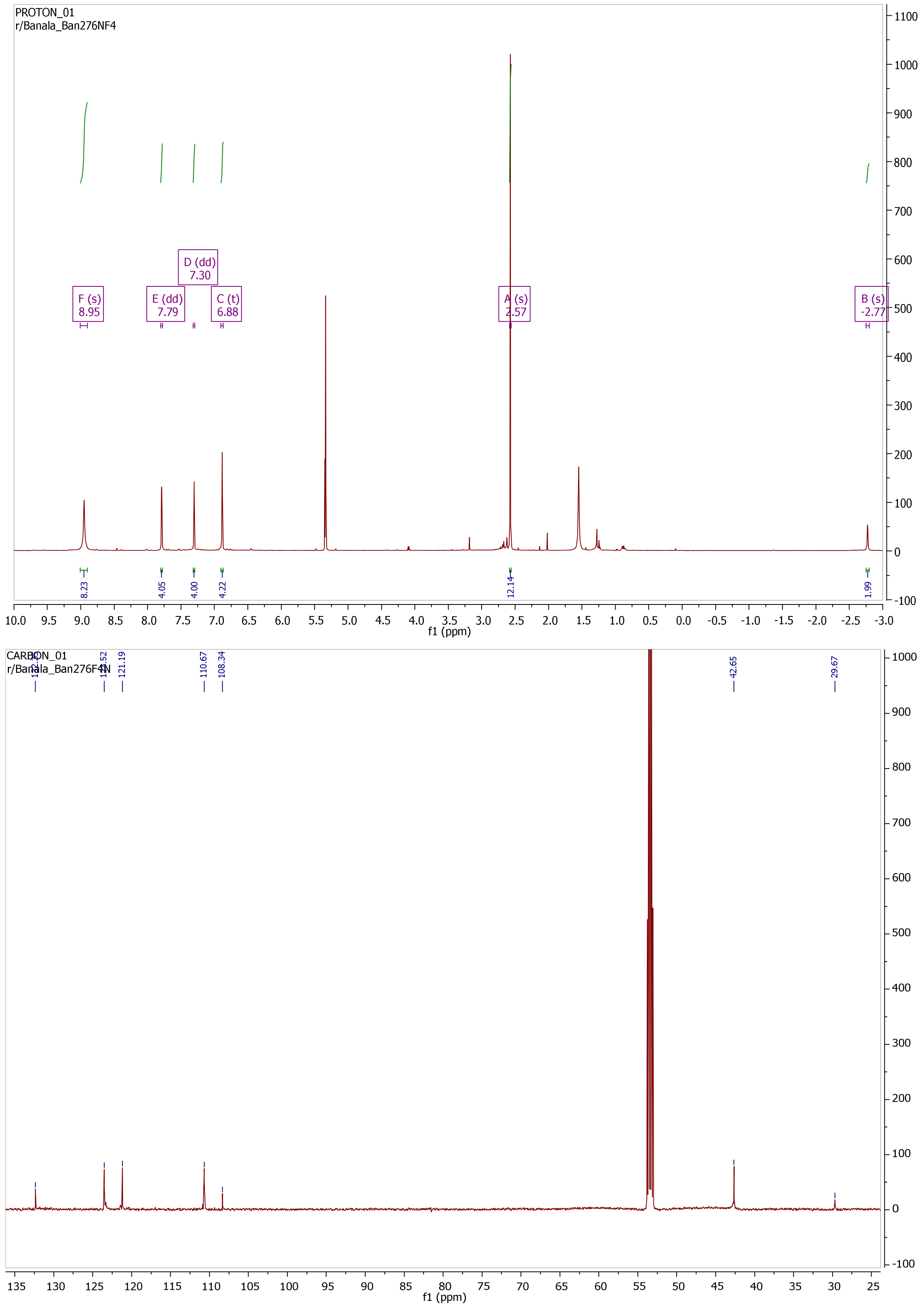

Fig S23: ^{1}H and ^{13}C NMR (600 & 125 MHz, CD_2Cl_2) of polar fraction **F4** of **3c-2H**

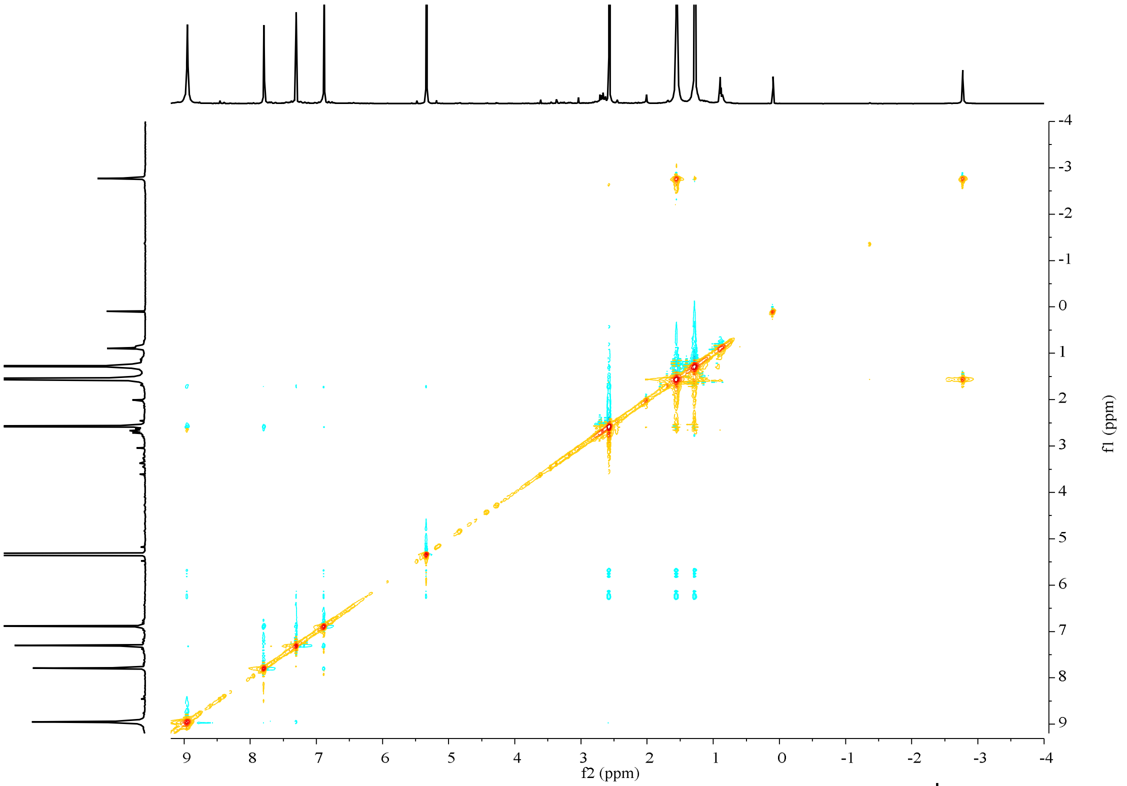

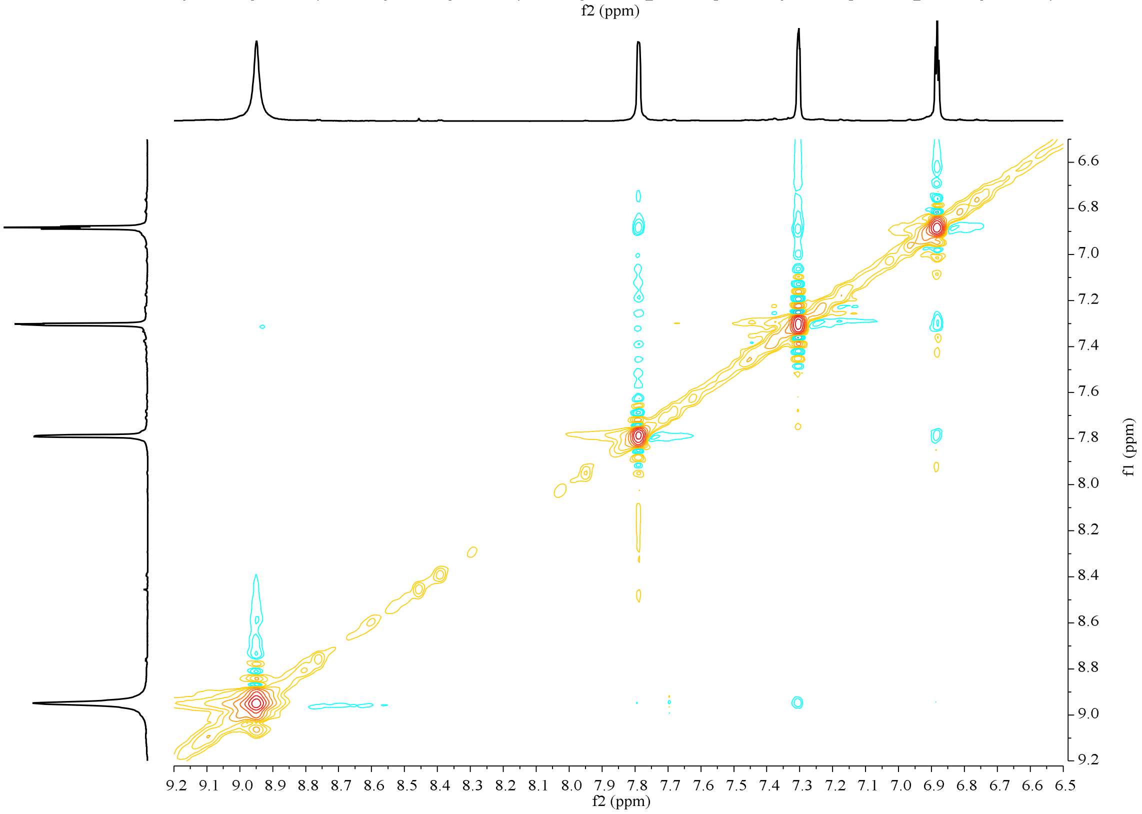

Fig S24: NOESY spectrum (in CD_2Cl_2, 600MHz) of polar fraction **F4** of **3c-2H,** bottom expanded aromatic region.

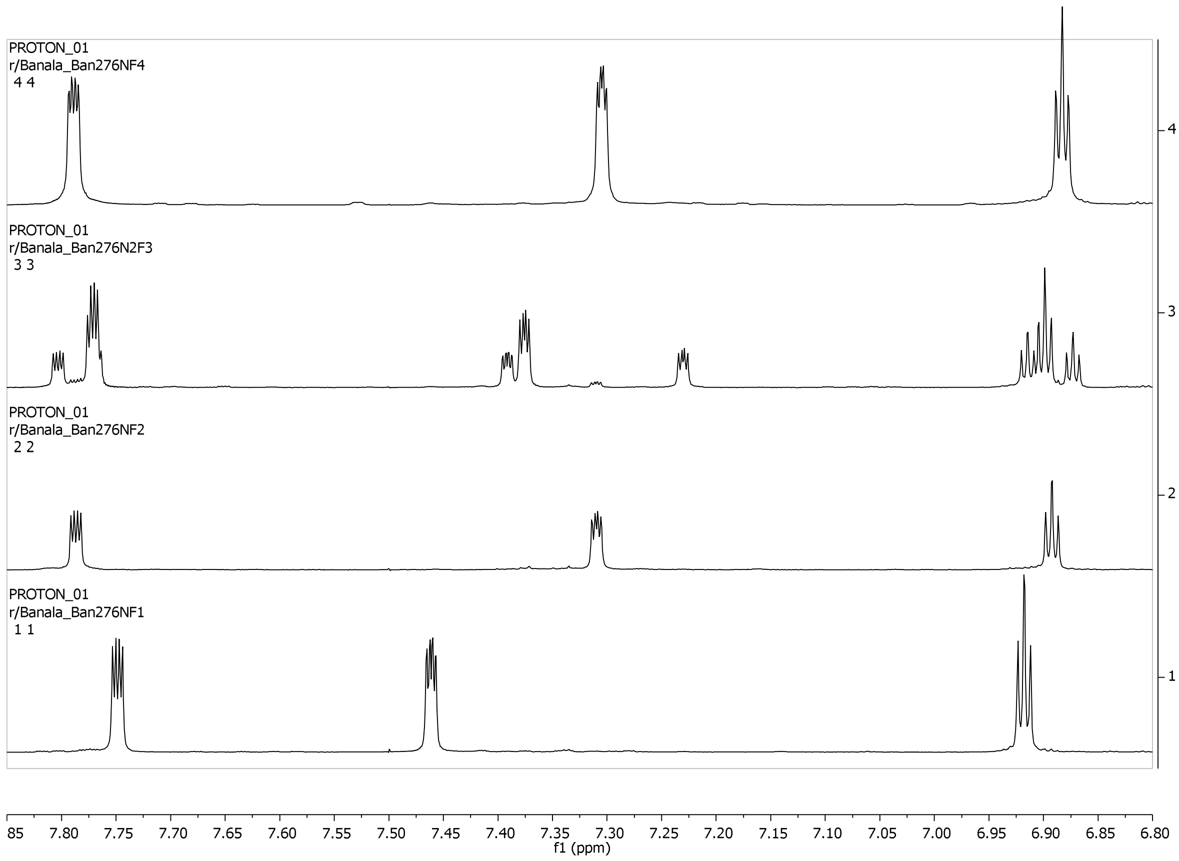

Fig S25: Overlay of expanded pyrrolic-protons region (^{1}H-NMR spectra, in CD_2Cl_2, 600MHz) of 4 atropisomeric fraction of **3c-2H**(F4 to F1, from top to bottom).

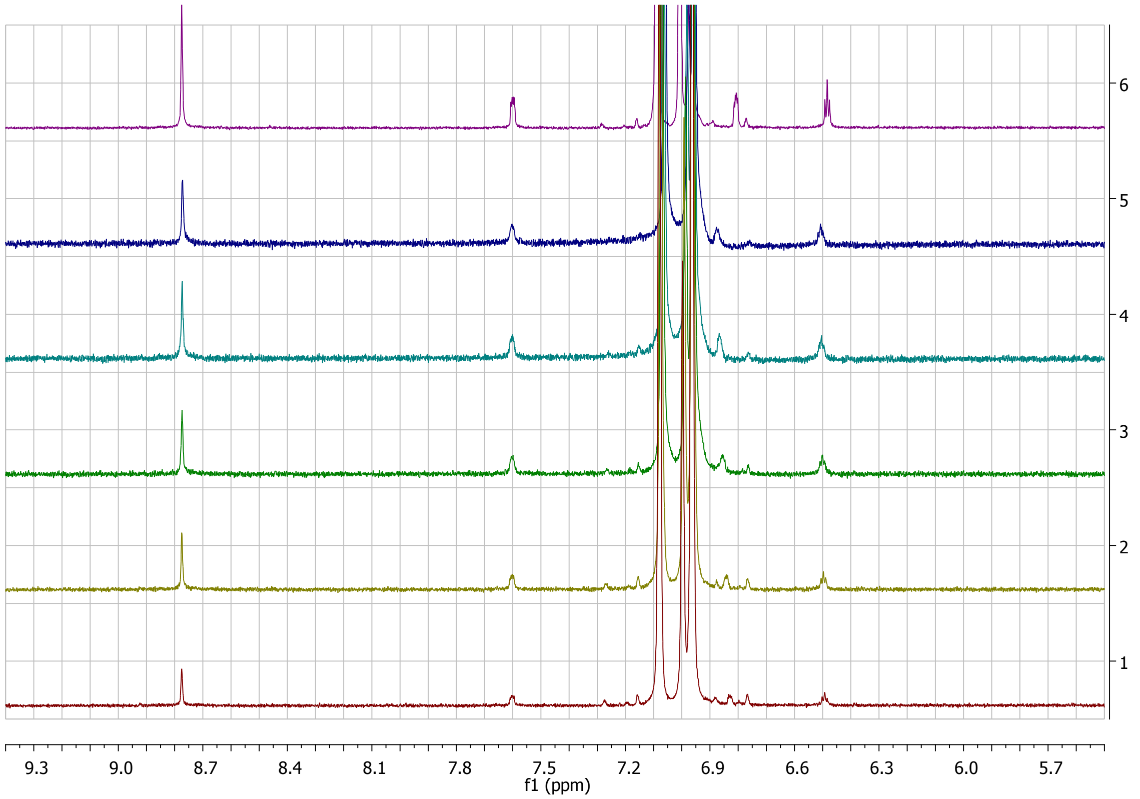

Fig S26. Variable temperature ^{1}H NMR (400 MHz, toluene-d8) of **3c-2H-F1**: From bottom to top [25 °C (1), 40°C (2), 50°C (3), 60°C (4), 70°C (5), to 80°C (6)]; note no change in peak at δ 8.8 ppm.

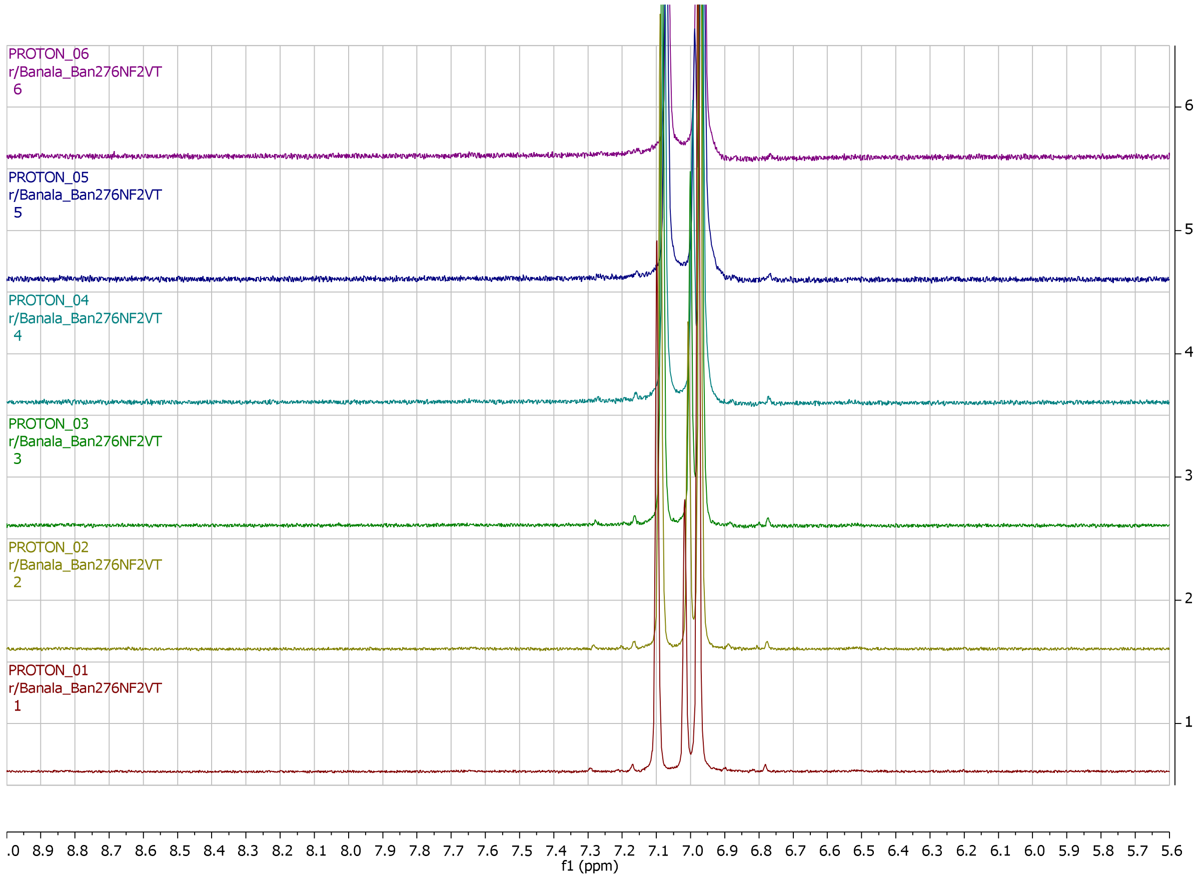

Fig S27: Variable temperature ^{1}H NMR (600 MHz, toluene-d8) of **3c-2H-F2**: From bottom to top [25 °C (1), 40°C (2), 50°C (3), 60°C (4), 70°C (5), 80°C (6)]. The fraction 2 is sparingly soluble, hence not showing any intense peaks for a clear change.

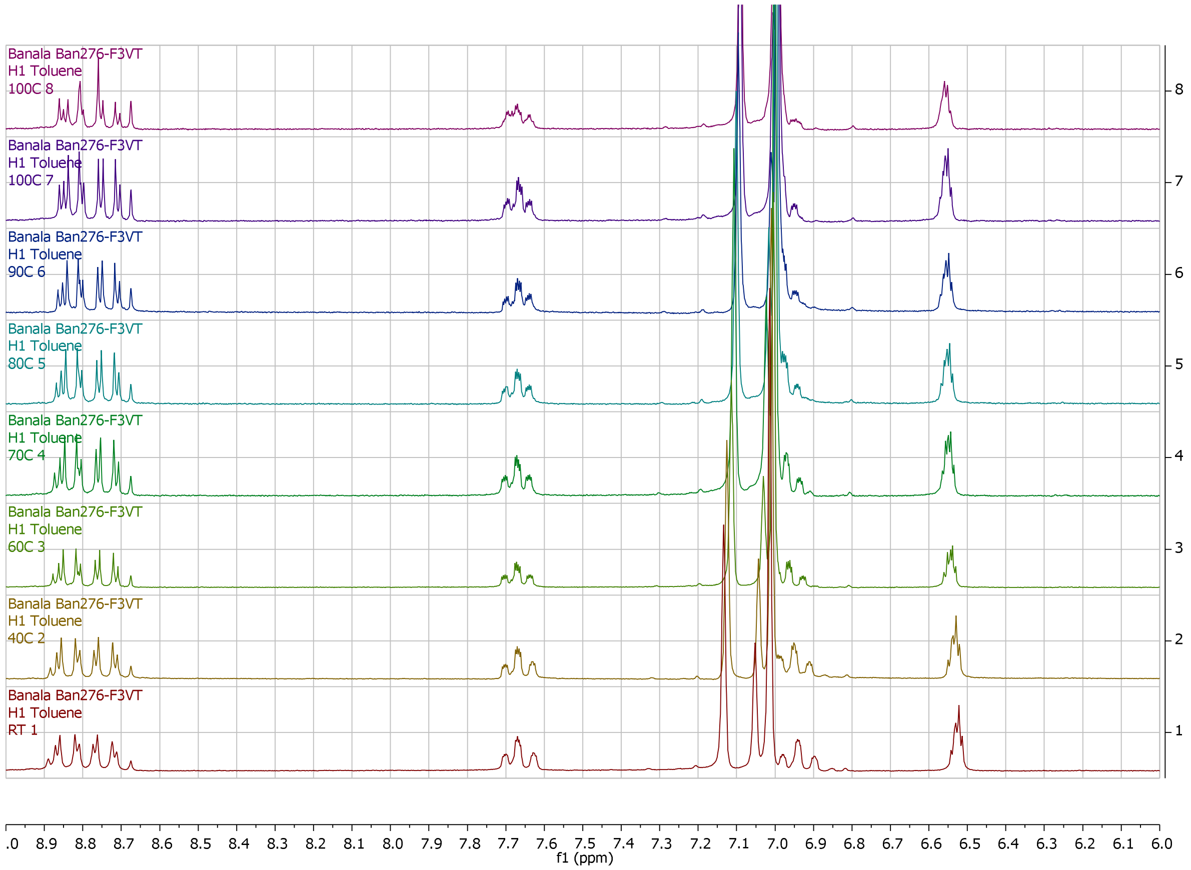

Fig S28: Variable temperature ^{1}H NMR (600 MHz, toluene-d8) of **3c-2H-F3**, for up to 100 °C. From bottom to top [25 °C (1), 40°C (2), 60°C (3), 70°C (4), 80°C (5), 90°C (6), 100°C (7), at 100°C, 15 min (8)].

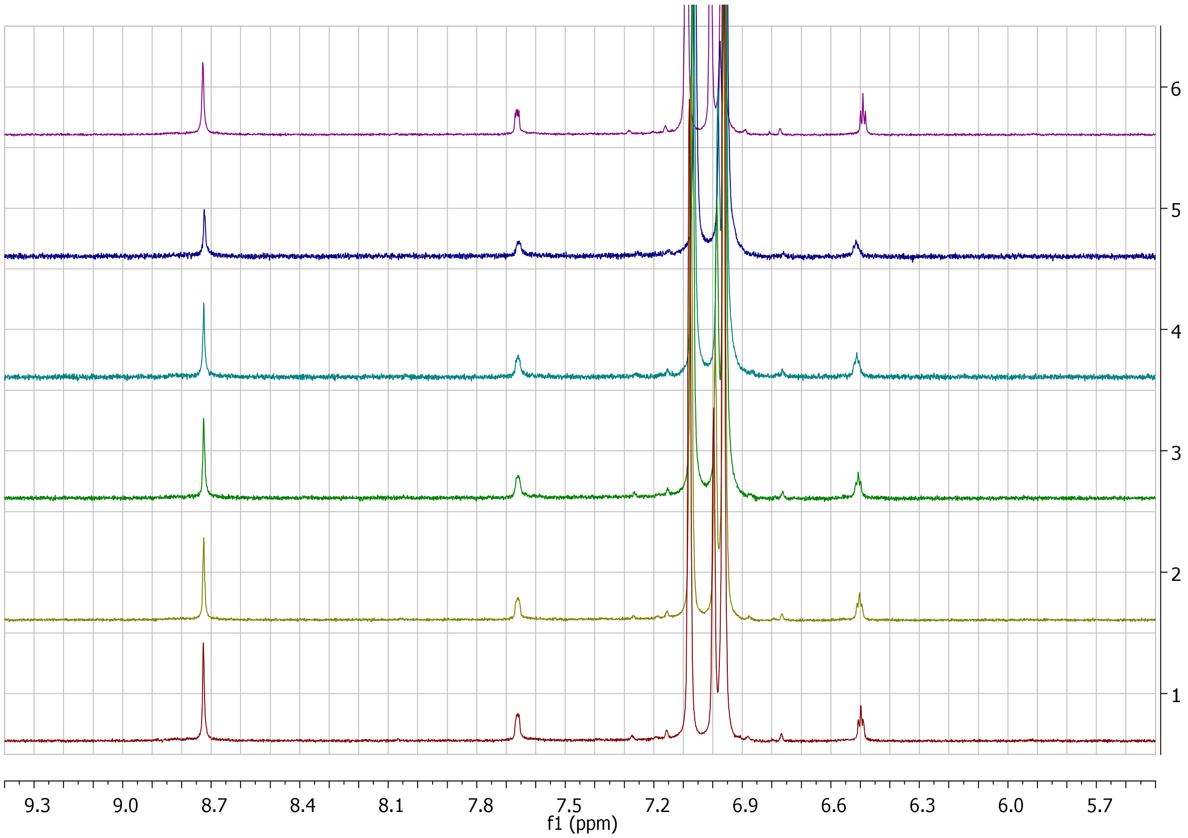

Fig S29. Variable temperature ^{1}H NMR (400 MHz, Toluene-d8) of **3c-2H-F4**: From bottom to top [25 °C (1), 40°C (2), 50°C (3), 60°C (4), 70°C (5), to 80°C (6)]; note no change in peak at δ 8.72 ppm.

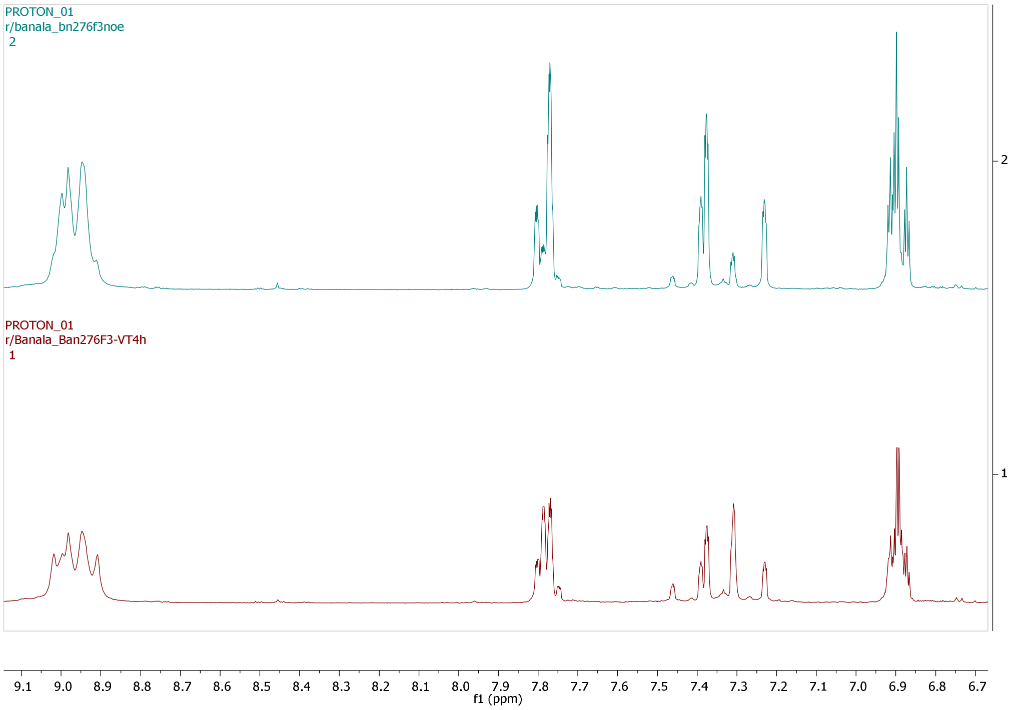

Fig 30-1: The aromatic region [1]**H-NMR** spectra (in CD_2Cl_2, 600MHz) of **3c-2H-F3** for heating at 100 °C, 4h; before heating (top) and (bottom) after heating.

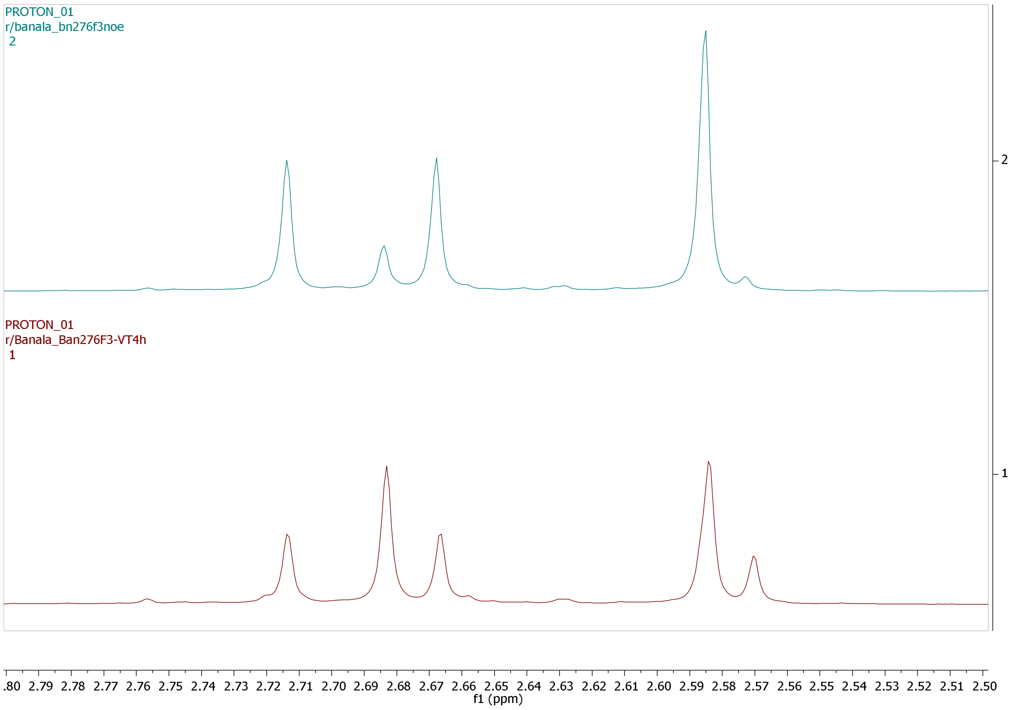

Fig S30-2: The aliphatic region [1]**H-NMR** spectra (in CD_2Cl_2, 600MHz) of fraction **3c-2H-F3** for heating at 100 °C for 4h; before heating (top) and (bottom) heating.

B) NMR Spectra: *meso* (*N*-Boc-pyrrol-2-yl) porphyrin (3a-2H)

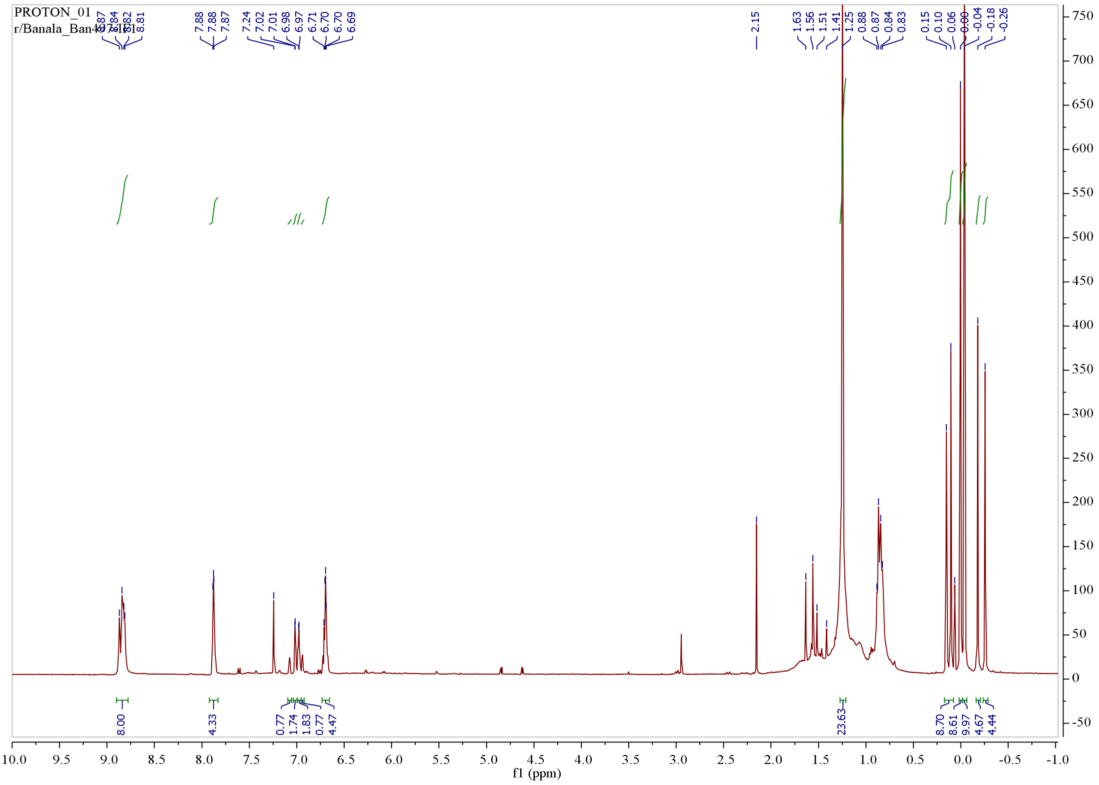

Fig S31: ^{1}H NMR (400 MHz, $CDCl_3$) of **3a-2H**

C) NMR Spectra: *meso* (*N*-Cbz pyrrol-2-yl) porphyrin (3b-2H)

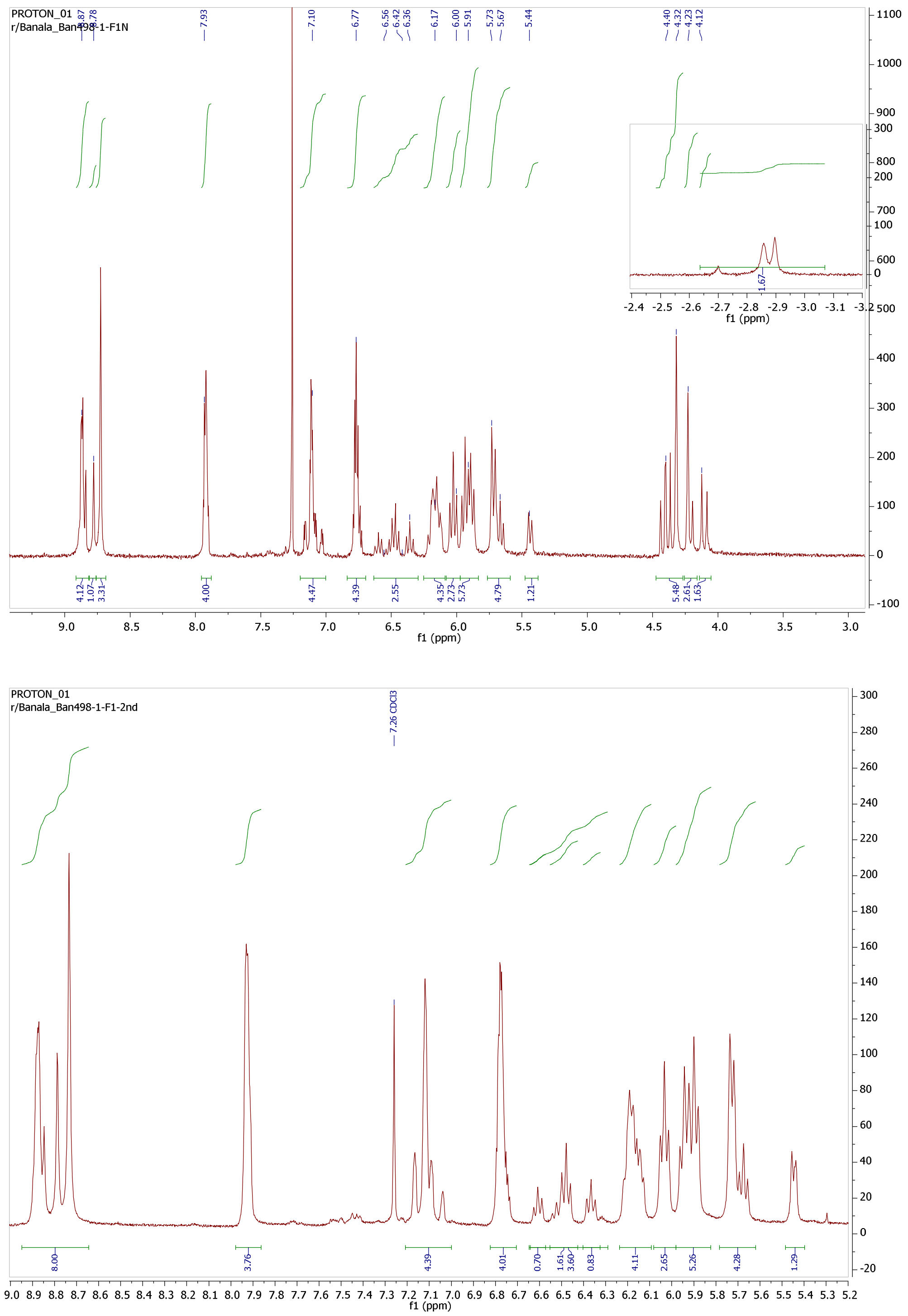

Fig S32: Top: ^{1}H NMR (400 MHz, $CDCl_3$) of **3b-2H** (inset: NH region); Bottom: Expanded aromatic region of **3b-2H**

D) NMR Spectra: *meso* (*N*-tosyl pyrrol-2-yl) porphyrin (3d-2H)

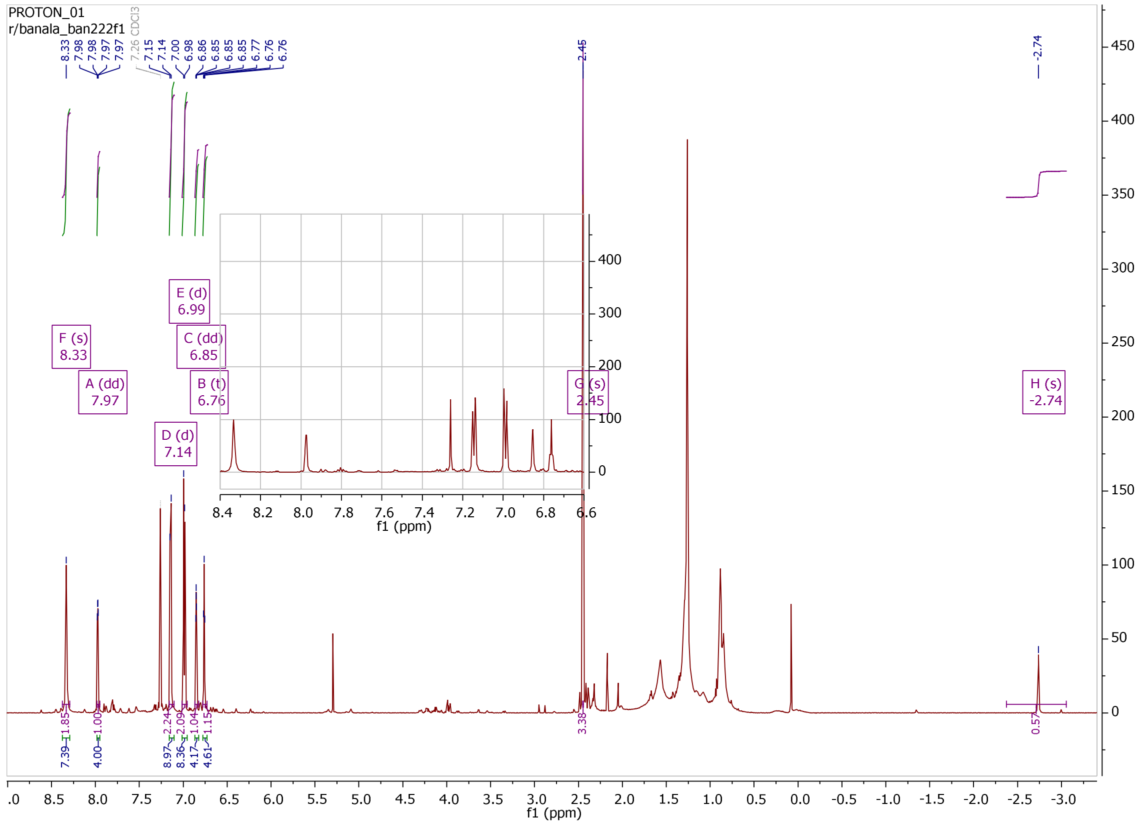

Fig S33-1: ^{1}H NMR (600 MHz, in $CDCl_3$) of apolar fraction 1 of **3d-2H**

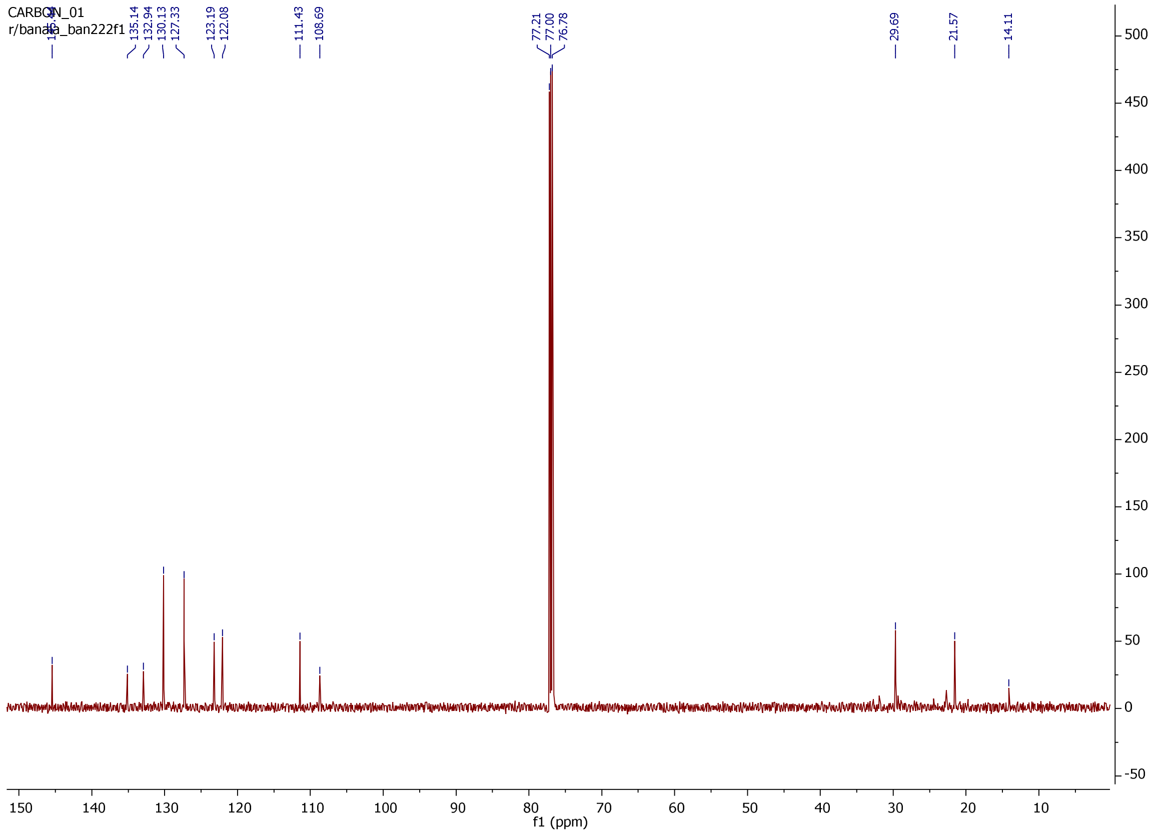

Fig S33-2: ^{13}C NMR (125 MHz, in $CDCl_3$) of apolar fraction 1 of **3d-2H**

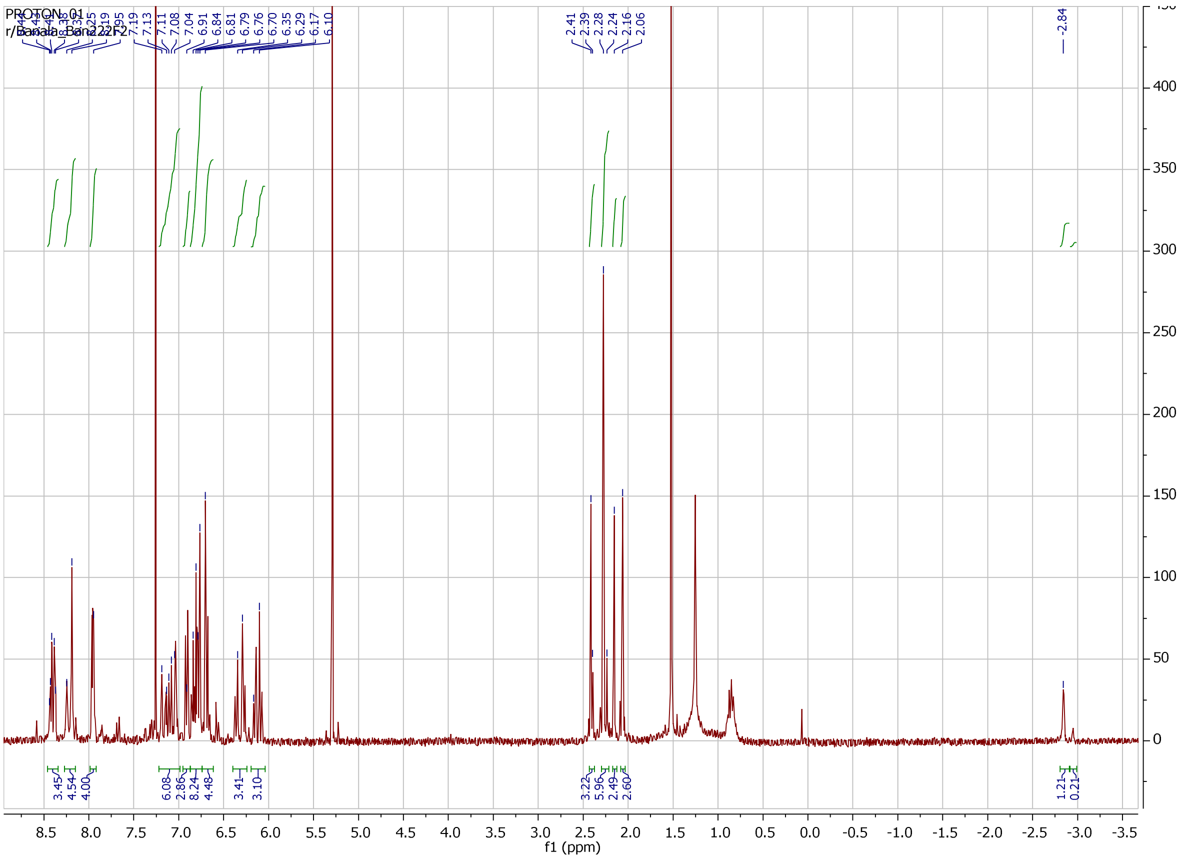

Fig S34: ^{1}H NMR (400 MHz, CD_2Cl_2) of polar fraction F2 of **3d-2H**

E) NMR Spectra: *meso* (*NH* pyrrol-2-yl) porphyrin (3e-2H)

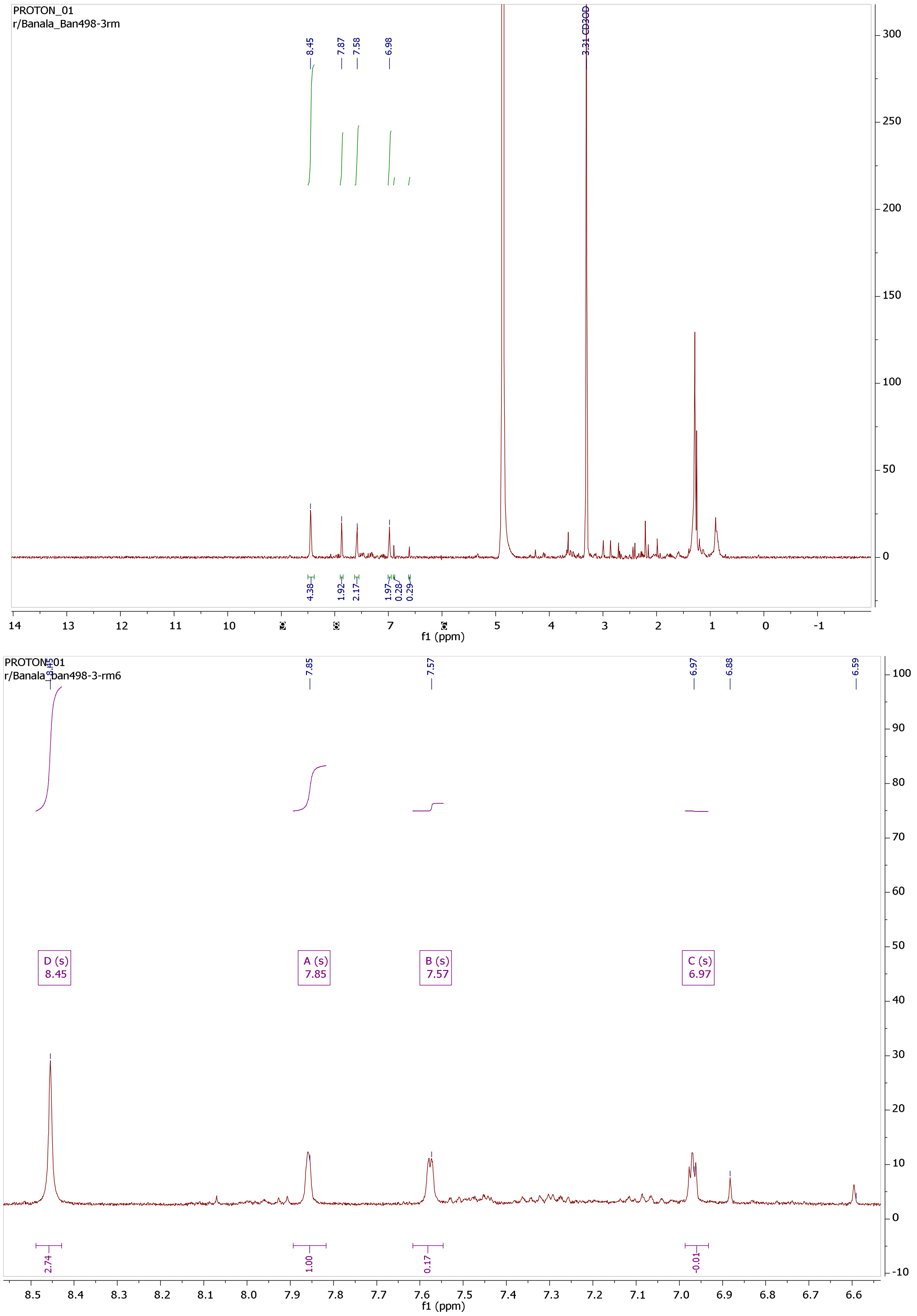

Fig S35: ^{1}H NMR (600 MHz, CD_3OD) of tetra (*NH*-pyrrol-2-yl) porphyrin **3e-2H** (of reaction mixture); Top: freshly prepared; Bottom (showed only aromatic region): after storing 3 months in CD_3OD in dark at room temperature.

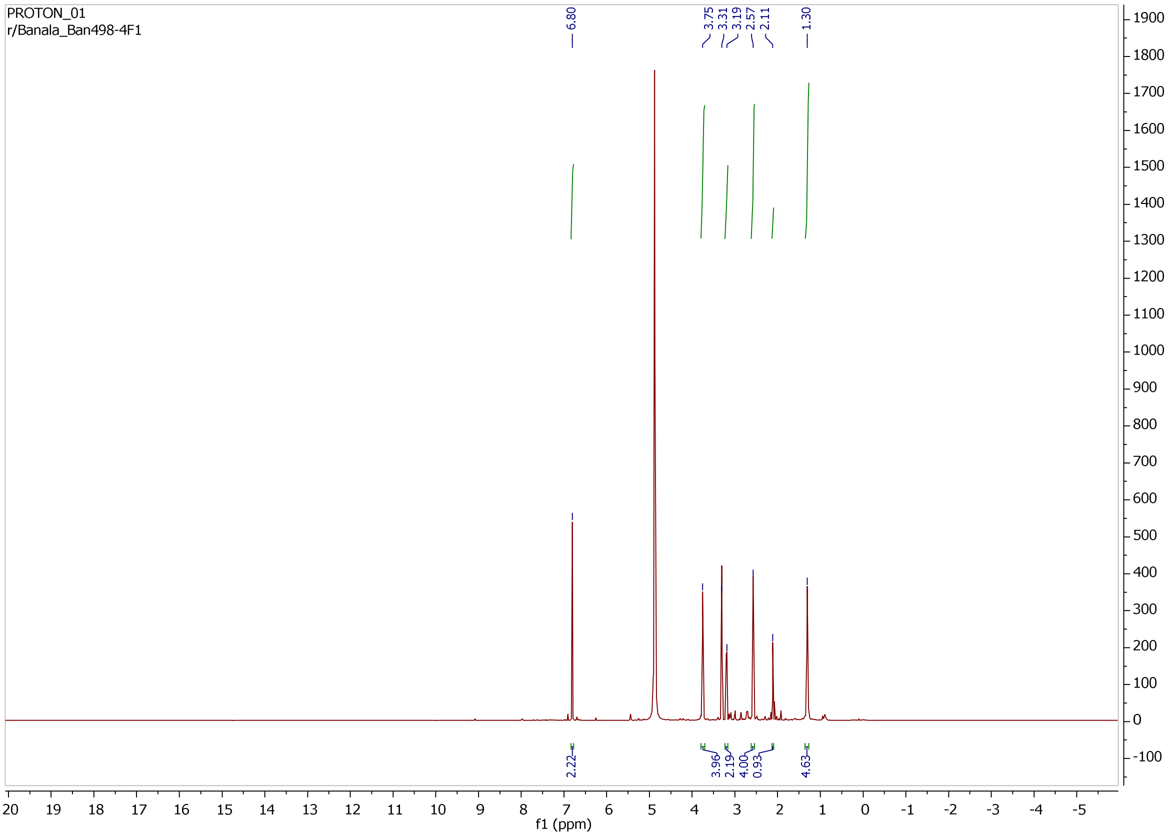

Fig S36: ^{1}H NMR (600 MHz, CD_3OD) of tetra (*NH*-pyrrol-2-yl) porphyrin **3e-2H** (after chromatographic purification, product found to be protonated)

7. Mass Spectra

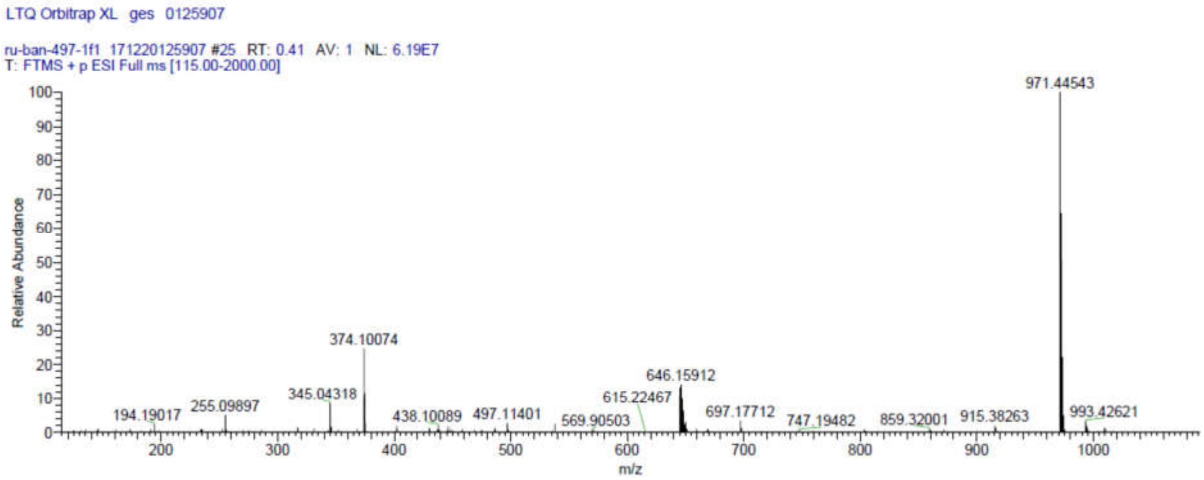

Fig S37: High Resolution ESI MS (positive ion) of the (*N*-Boc-pyrrol-2-yl)-porphyrin (**3a-2H)**

(calc. $C_{56}H_{59}N_8O_8^+$: 971.44504, m/z_{found} = 971.44543 (100%, $[M+H]^+$)

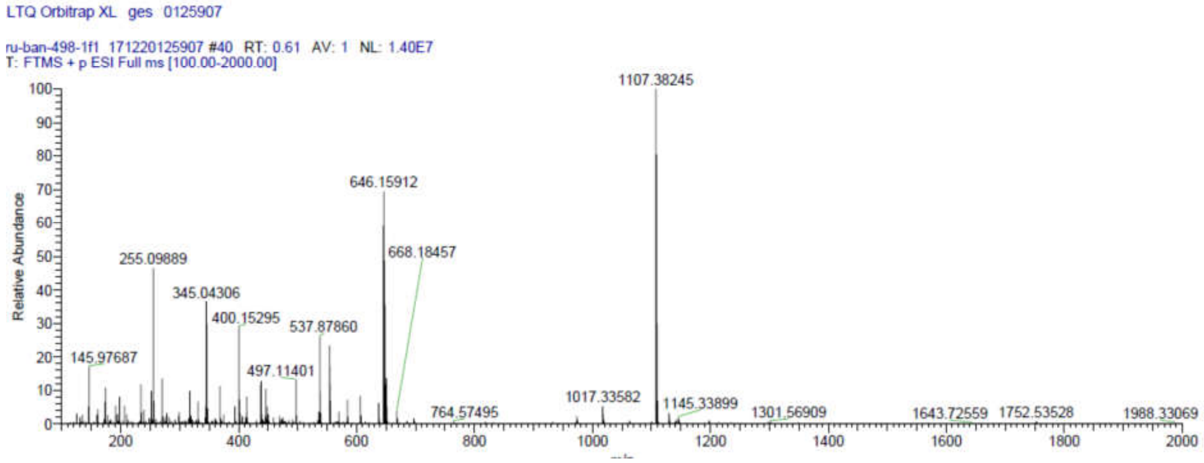

Fig S38: High Resolution ESI MS (positive ion) of the (*N*-*C*bz-pyrrol-2-yl)-porphyrin (**3b-2H)**

(calc. $C_{68}H_{51}N_8O_8^+$: 1107.38244, m/z_{found} = 1107.38245 (100%, $[M+H]^+$)

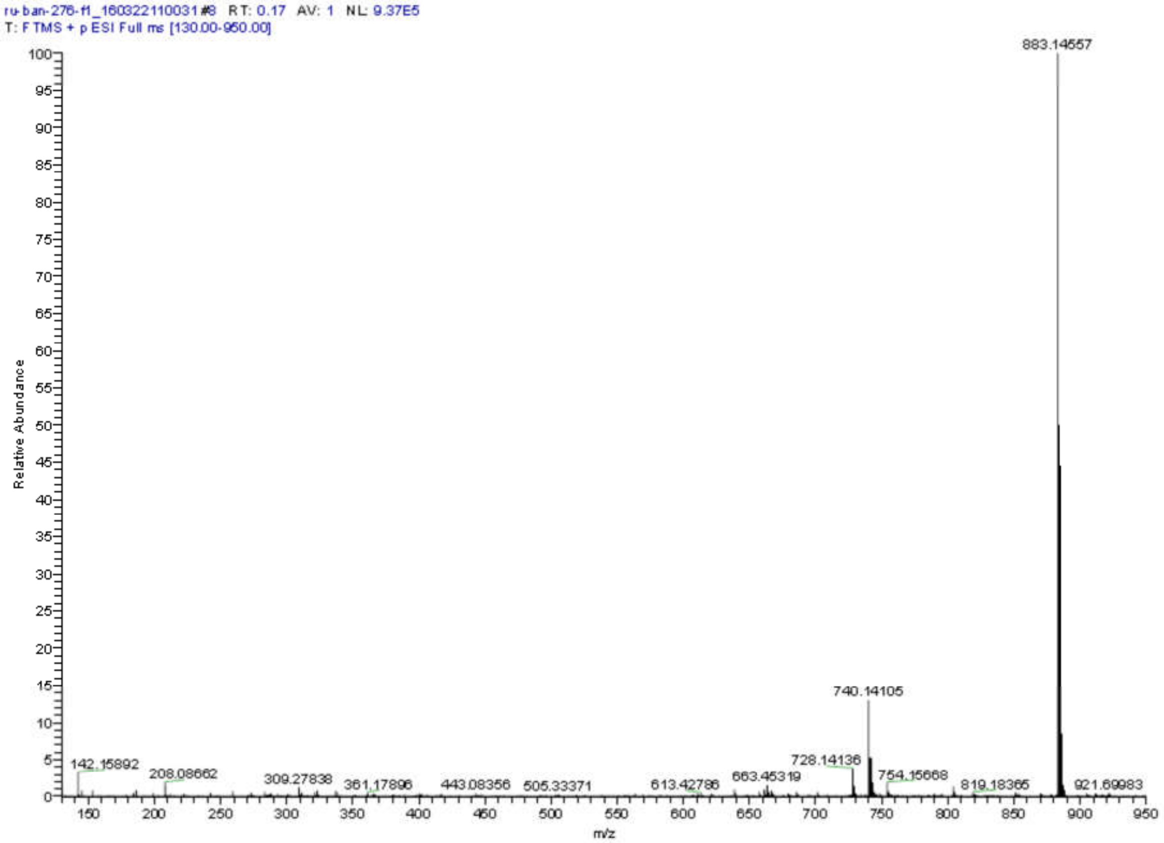

Fig S39: High Resolution ESI MS (positive ion) of apolar fraction F1 of **3c-2H**

(calc. $C_{40}H_{35}N_8O_8S_4^+$: 883.14552, m/z_{found} = 883.14539 (76%, $[M+H]^+$)

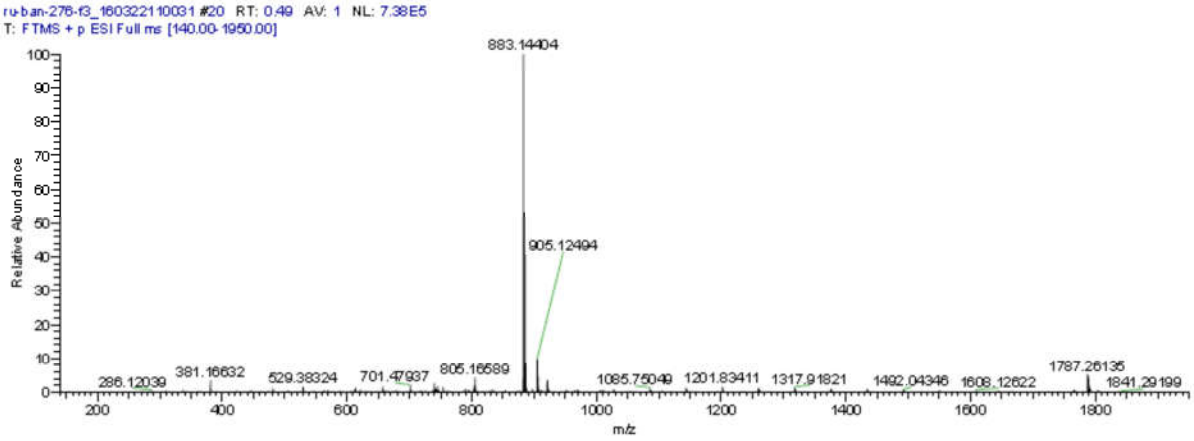

Fig S40: High Resolution ESI MS (positive ion) of middle polar isomer, F3 of **3c-2H**. (calc. $C_{40}H_{35}N_8O_8S_4^+$: 883.14552), m/z_{found} = 883.14404 (100%, $[M+H]^+$)

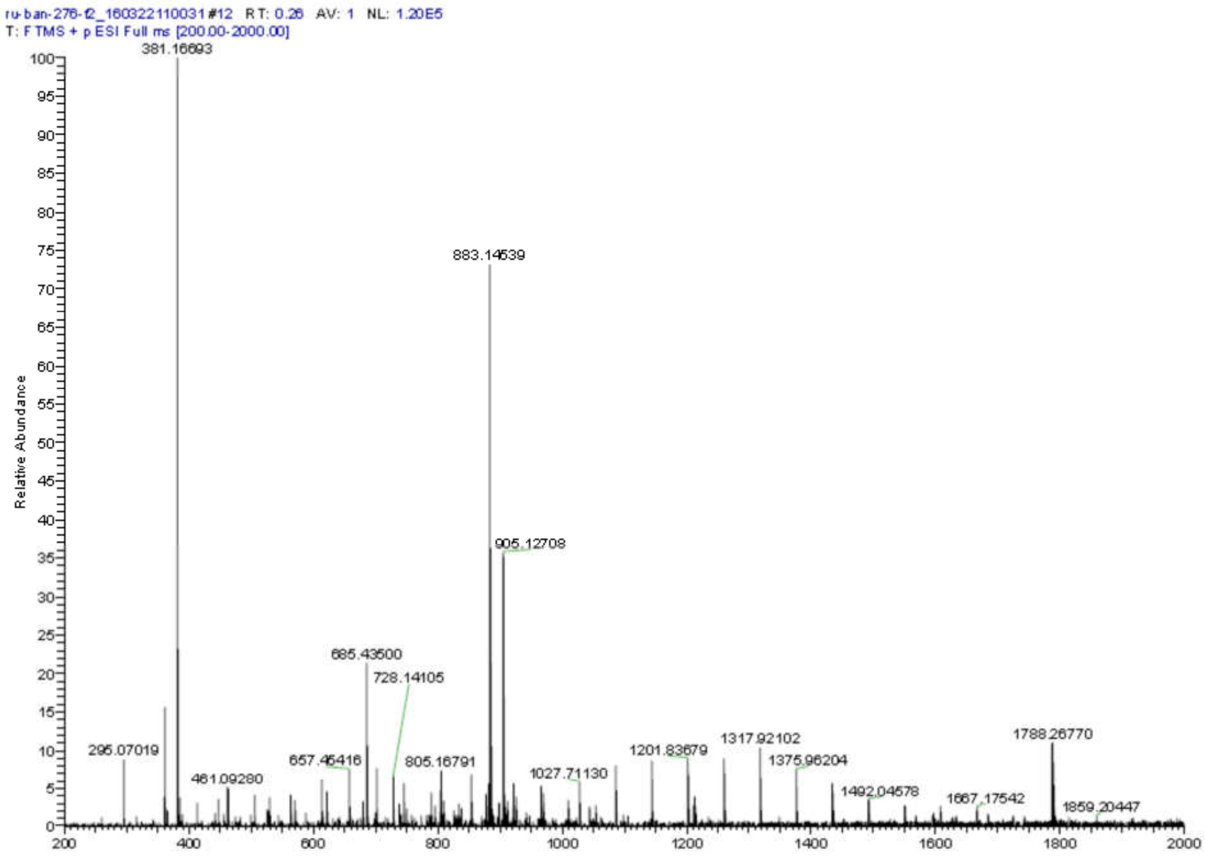

Fig S41: High Resolution ESI MS (positive ion) of middle polar isomer, F2 of **3c-2H**

(calc. $C_{40}H_{35}N_8O_8S_4^+$: 883.14552, m/z_{found} = 883.14539 (76%, $[M+H]^+$)

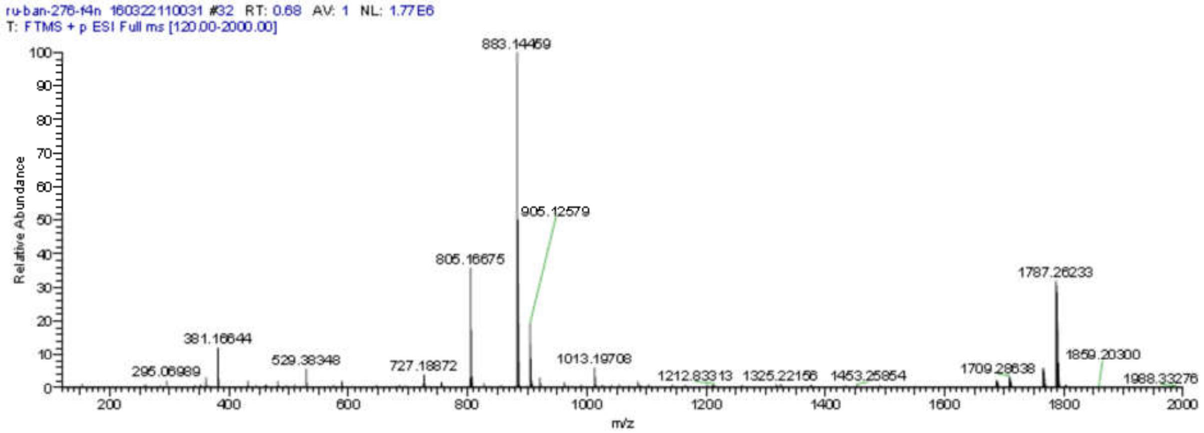

Fig S42: High Resolution ESI MS (positive ion mode) of polar isomer, F4 of **3c-2H**; (calc. $C_{40}H_{35}N_8O_8S_4^+$: 883.14552, m/z_{found} = 883.14559 (76%, $[M+H]^+$)

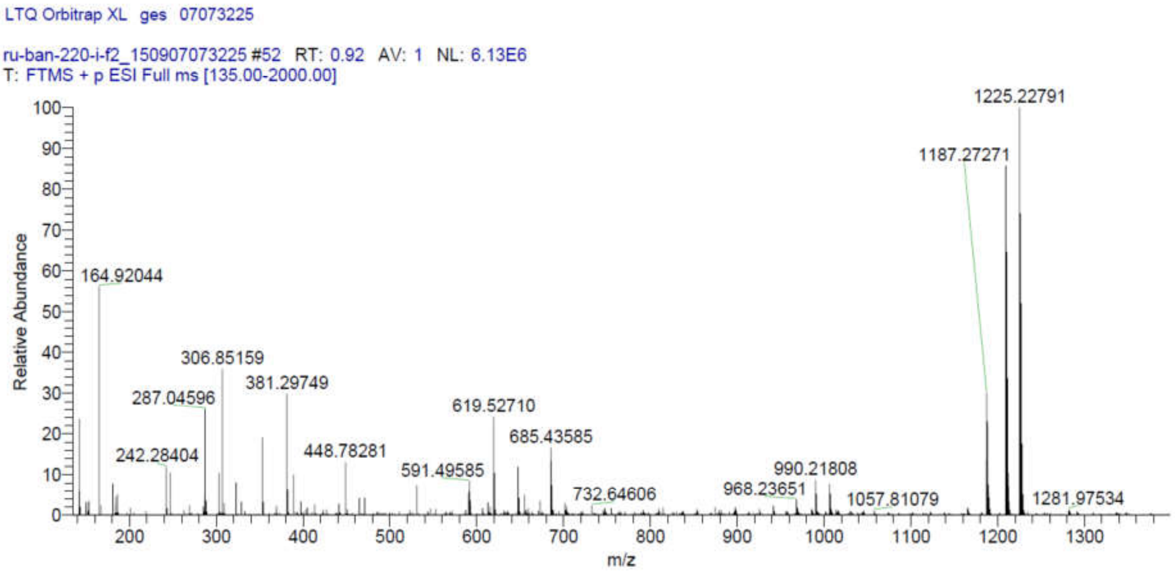

Fig S43: High Resolution ESI MS (positive ion) of the (*N*-Tosyl-pyrrol-2-yl)-porphyrin (**3d-2H)**

(calc $C_{64}H_{51}N_8O_8S_4^+$: 1187.27072, found: 1187.27271 (35%, $[M+H]^+$); 1225.22791 $[M+K]^+$)

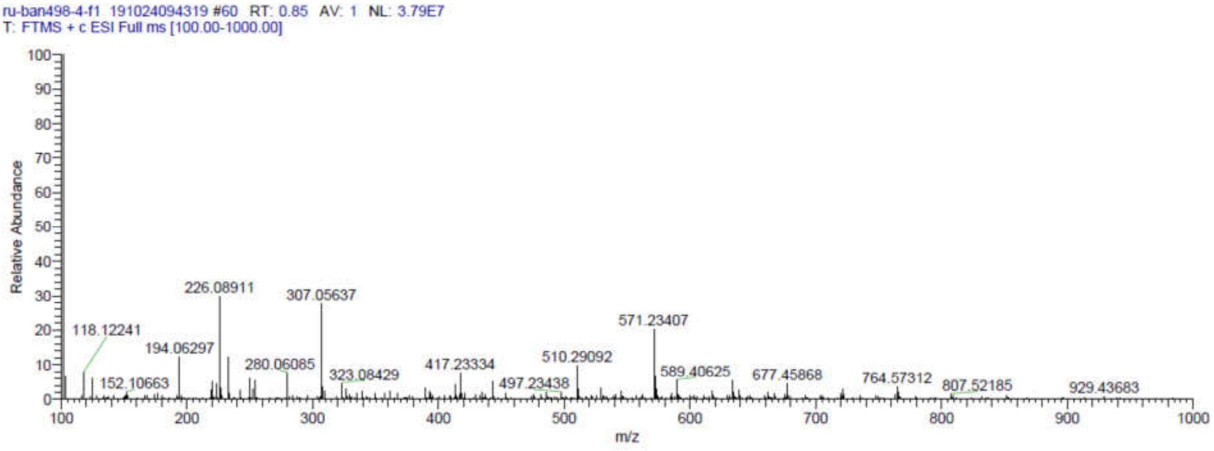

Fig S44: High Resolution ESI MS (positive ion) of the meso (*NH*-pyrrol-2-yl)-porphyrin (**3e-2H)**

(calc $C_{36}H_{27}N_8^+$: 571.23532, m/z_{found} = 571.23407 ($[M+H]^+$)
